# Solvent Effects and Aggregation Phenomena Studied by Vibrational Optical Activity and Molecular Dynamics: The Case of Pantolactone

**DOI:** 10.1021/acs.jpcb.0c01483

**Published:** 2020-05-12

**Authors:** Simone Ghidinelli, Sergio Abbate, Jun Koshoubu, Yasuyuki Araki, Takehiko Wada, Giovanna Longhi

**Affiliations:** † Dipartimento di Medicina Molecolare e Traslazionale, Università di Brescia, Viale Europa 11, 25123 Brescia, Italy; ‡ Istituto Nazionale di Ottica (INO), CNR, Research Unit of Brescia, c/o CSMT, Via Branze 45, 25123 Brescia, Italy; § JASCO Corporation, 2967-5 Ishikawa-machi, Hachioji, Tokyo 192-8537, Japan; ∥ Institute of Multidisciplinary Research for Advanced Materials, Tohoku University, 2-1-1 Katahira, Aoba-ku, Sendai, Miyagi 980-8577, Japan

## Abstract

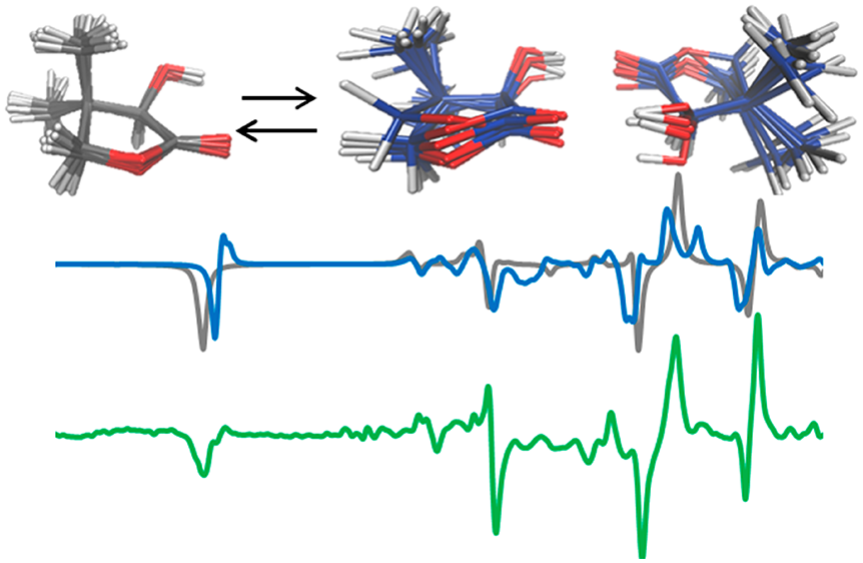

Raman and Raman optical activity (ROA), IR, and vibrational circular dichroism (VCD) spectra of (*R*)- and (*S*)-pantolactone have been recorded in three solvents. ROA has been employed on water and DMSO solutions, VCD on DMSO and CCl_4_ solutions. In the last solvent, monomer–dimer equilibrium is present. Due to the low conformational flexibility of the isolated molecule and to the possibility of aggregation, this compound has been used here to test different protocols for computation of the spectroscopic responses taking into account solvent effects. Molecular dynamics (MD) simulations have been carried out together with statistical clustering methods based on collective variables to extract the structures needed to calculate the spectra. Quantum mechanical DFT calculations based on PCM are compared with approaches based on different representations of the solvent shell (MM or QM level). Appropriate treatment of the solvent permits obtaining of good band-shapes, with the added advantage that the MD analysis allows one to take into account flexibility of dimeric structures justifying the broadness of observed bands and the absence of intense VCD couplets in the carbonyl and OH stretching regions.

## Introduction

Chiroptical spectroscopies, particularly considering vibrational optical activity (VOA), permit gaining of information not only concerning configuration assignment but also with respect to conformations and intermolecular interactions: solute–solvent interplay influences the solute conformational landscape; solute–solute interactions may originate aggregation effects. On these aspects, recent literature has proposed many protocols to deal with solute–solvent interactions using geometrical initial conditions for optimization based on chemical intuition or by modeling solvent interactions with the aid of molecular dynamics (MD) classical or ab initio simulations. Good results were obtained with the “cluster in a liquid” approach^1^ adopted in particular to explain vibrational circular dichroism (VCD) spectra recorded in organic solvents;^1,2^ however solvents with donor capability like methanol seem particularly difficult to treat satisfactorily. The use of molecular dynamics (MD) simulations within a box of solvent molecules seems mandatory in water solutions, usually studied with Raman optical activity (ROA) spectroscopy.^3−8^ Recently, the use of molecular simulations has been exploited to calculate VCD response directly from the trajectories through the cross-correlation function of the electric and magnetic dipole moments as proposed in ref (9). Increasingly demanding simulative methods have been adopted: classical dynamics,^10^ QM/MM molecular dynamics calculations,^11^ and combined MD/ab initio DFT methods.^12−14^ The latter approach based on quantum mechanics is quite interesting, avoiding approximate models to calculate magnetic dipole transition moment, but is quite demanding in terms of computational efforts and prohibitive if long simulations are needed to avoid the initial condition dependence.

In the following, we critically discuss new VOA data regarding (*R*)- and (*S*)-pantolactone explicitly taking into account solvent molecules with a detailed analysis of classical MD results. This molecule possesses few and to some extent restricted conformational degrees of freedom, so one cannot expect great differences due to different conformer populations such that the observed spectral variations from solvent to solvent can be attributed either to subtle solute–solvent interactions or to aggregation phenomena. For this reason, this molecule may be considered a good benchmark to test the usefulness of classical MD simulation to obtain a number of representative snapshots and subsequent QM calculation of the chiroptical responses with the usual magnetic field response theory developed for VCD^15−17^ and for ROA.^18,19^

We will examine the case of pantolactone in water and DMSO, solvents quite prone to form H-bonds. The comparison of the different calculations with experimental data (ROA for water, ROA and VCD for DMSO) will show to what extent the MD–QM treatment is appropriate. The same compound can be dissolved in CCl_4_: IR, NMR,^20^ and ORD literature data^21,22^ indicate that this compound forms dimers in CCl_4_ solution; a dimerization constant was evaluated,^20^ and also the two OR values of the monomeric and dimeric forms were deduced.^22^ In this work, we have measured IR and VCD spectra in various spectroscopic regions: such spectra contain rather informative data in the C=O and OH stretching regions, which potentially carry information on aggregation. Also in this case, following our recent experience with MD and QM/MM calculations,^23^ we have investigated pantolactone dimer formation.

## Experimental Section

The (*R*)- and (*S*)-pantolactone enantiomers (α-hydroxy-β,β-dimethyl-γ-butyrolactone) were purchased from ACROS Organics and Tokyo Chemical Industry. These compounds have been used without further purification. IR and VCD spectra have been recorded on 49 mM CCl_4_ and 410 mM DMSO-*d*_6_ solutions, using a JASCO FVS-6000 VCD spectrometer. A fixed path length 200 μm cell with BaF_2_ windows and 2 mm cell with CaF_2_ windows were used for 2000–850 cm^–1^ and 4000–2650 cm^–1^ spectral regions. The spectral resolution values for 2000–850 cm^–1^ and 4000–2650 cm^–1^ spectral regions are 4 and 8 cm^–1^ respectively. The spectra for 2000–850 cm^–1^ and 4000–2650 cm^–1^ spectral regions have been measured using MCT detector and InSb detector with a collection time of 5 and 2 h, respectively. The presented spectra are solvent subtracted.

The ROA experiments were carried out using a newly developed apparatus based on incident circularly polarized (ICP) backscattering ROA.^24−26^ The excitation light source is a continuous wave green laser (COHERENT, Genesis MX, 532 nm, max 1 W). The linearly polarized excitation light is converted to the circularly polarized light using a quartz λ/4 wave plate. The left and right circularly polarized light can be switched by inserting two λ/2 wave plates. The backscattered light from the liquid sample in a rectangular quartz cell was collected by a lens and passed an edge filter. Finally, the scattered light was focused on a bundle fiber, introduced into a high-throughput spectrometer, and then detected by a Peltier cooled CCD. Raman and ROA spectra have been recorded on 873 mM water and 410 mM DMSO-*d*_6_ solutions, using our ICP backscattering ROA spectrometer. The spectral resolution for the 2000–200 cm^–1^ spectral region is 4 cm^–1^. The collection times for water solution and DMSO-*d*_6_ solution were 4 and 16 h, respectively. The presented spectra are solvent subtracted.

## Computational Methods

### Conformational Search

A molecular mechanics conformational search has been undertaken for the monomer and for the dimer, confirming the results by Beratan et al.^21^ Subsequent analysis has been conducted also using the CREST program^27^ which is based on a semiempirical tight-binding quantum chemistry method (GFN2). The use of metadynamics coupled with genetic z-matrix crossing approach allows for a thorough conformational search. For the conformational sampling of the dimer, the CREST program was used in the NCI modality.^27^

### Molecular Dynamics Simulations

MD simulations were carried out using the GROMACS-2016 package.^28^ Bonds with H atoms were constrained by the LINCS algorithm.^29^ The 10 Å cutoff value was used for nonbonded interactions, and long-range electrostatic interactions were handled with the PME scheme.^30^ The stochastic velocity rescaling thermostat^31^ with 0.1 ps time constant and the Parrinello–Rahman pressure coupling protocol^32^ with 2 ps time constant were used. The general Amber force field (GAFF)^33^ was used for all simulations. (*R*)-Pantolactone atomic charges have been calculated at the HF-6.31G* level through the RESP^34^ protocol implemented in Amber-Tools16^35^ package. The parameters reported in the Fox and Kollman work^36^ have been used for CCl_4_ and DMSO solvents. TIP3P model was used for water solvent. Three simulations have been conducted, one for each solvent, at 300 K and 1 atm. In each simulation two molecules of (*R*)-pantolactone have been considered in cubic solvent boxes with 750 CCl_4_, 870 DMSO, 3660 water molecules, respectively. Preliminary tests have been carried out considering various initial distance values between the two molecules of pantolactone within the box. In water and DMSO the two molecules throughout the simulation are far apart from each other; that is to say they may be considered in monomeric form. In CCl_4_, instead, the dimeric form seems to prevail. Water and DMSO solutions have been simulated for 60 ns, and CCl_4_ solution has been simulated for 150 ns.

### Trajectory Analysis

Analysis has been conducted on frames taken every 10 ps. H-bonds have been evaluated using VMD software.^37^ Criteria for the formation of hydrogen bonds are donor–acceptor distance less than 3 Å and D–H–A angle less than 30°.

**Scheme 1:**
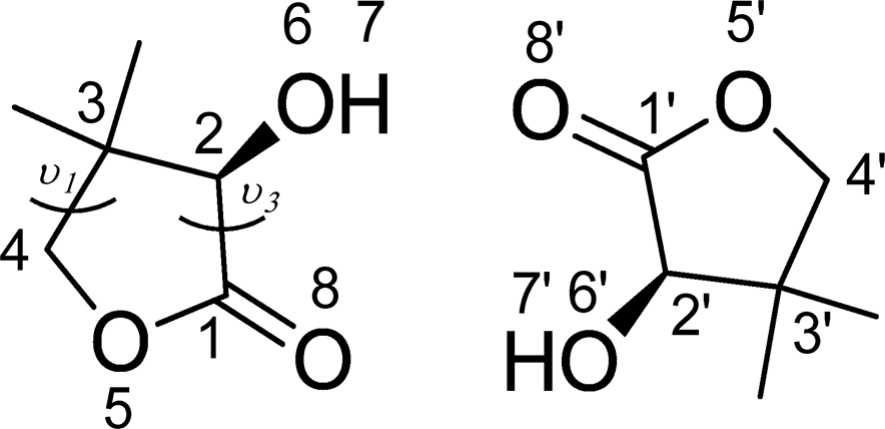
Structure and Atom Numbering of an (*R*)-Pantolactone Dimer

The frames of the trajectories have been clustered using Metagui3,^38^ a plugin of VMD software.^37^ Since during the simulations in water and DMSO no dimerization occurs, the two (*R*)-pantolactone molecules have been analyzed individually, as independent systems. Two collective variables (CVs), defined and implemented in the PLUMED^39^ modulus, have been chosen: hydroxyl torsion (atoms 7–6–2–1 in Scheme 1) and five-membered ring puckering phase *P*_θ_,^40^ which takes into account the degrees of freedom of the molecule ring. The definition of the puckering coordinates in ref (40) is based on the one given by Sato^41^ and Altona:^42^*Z*_*x*_ and *Z*_*y*_ are linear combination of two torsions ν_1_ and ν_3_ of the ring (see Scheme 1).
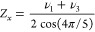

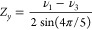
The phase *P*_θ_ and amplitude *A*_r_ of the pseudorotation is given by
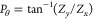
1
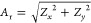
2Statistical analysis of the two CVs, OH torsion and *P*_θ_, suggested the presence of six principal groups of values; thus six clusters have been isolated using the k-medoids algorithm.^38,43^ Concerning the simulation in CCl_4_ solvent, the pantolactone molecules are in monomeric form during some time intervals, but they prevalently dimerize forming intermolecular hydrogen bonds. We adopted a 4 Å donor–acceptor distance as a threshold for distinguishing monomers from dimers. Monomeric structures have been analyzed as previously described. For dimers, three collective variables have been chosen: five-membered ring puckering phase for the first pantolactone (*P*_θ_A), five-membered ring puckering for the second pantolactone phase (*P*_θ_B), and a “pseudo”-six-membered intermolecular ring puckering phase (φ) evaluated according to the definition of Cremer and Pople^44^ considering the ring defined by atoms numbered as 6–1–8–6′–1′–8′ in Scheme 1. This intermolecular pseudopuckering phase appears to discriminate the different reciprocal orientations of the two pantolactone molecules better than other couples of dihedral angles like, 1–2–1′–2′, 8–6–8′–6′, 2–6–8′–1′, and 1–8–6′–2′ in Scheme 1. On the basis of the behavior of the three above-described CVs, 16 clusters have been grouped using the k-medoids algorithm. For all three simulations, the center of each cluster has been extracted with a 6 Å solvation shell.

### QM and QM/MM Calculations

All QM optimizations and frequency calculations of (*R*)-pantolactone were performed at the DFT B3LYP/6-311++G(2d,2p) level of theory with IEF-PCM approximation^45^ as implemented in the Gaussian 16^46^ program. Considering the structures obtained through the conformational search, the simple IEF-PCM implicit solvent scheme has been first adopted, and the population factors of the various conformers have been calculated from DFT energy values.

For the three solvents considered in this work, after obtaining structures by MD simulations and clustering, a two-layer ONIOM calculation has been conducted on the representative of each cluster: optimizations and frequency calculations were carried out treating (*R*)-pantolactone at the DFT B3LYP/6-311++G(2d,2p) level of theory, while the solvent layer (6 Å) was considered at the MM level with the same parameters used in the MD simulations, adopting electronic embedding and IEF-PCM. For brevity, we call this approach QM/MM. In addition, for water and DMSO solutions, on the same representative structures obtained by MD analysis, a 4 Å shell of solvent molecules has been treated at the same DFT, IEF-PCM level as pantolactone; hereafter this approach is called QM/QM.

Raman and ROA spectra have been calculated using the “two steps” procedure proposed by Cheeseman and Frisch:^47^ B3LYP/6-311++G(2d,2p) followed by B3LYP/aug-cc-pvdz. 6 cm^–1^ Lorentzian bandwidth has been applied to all vibrational transitions.

## Results and Discussion

### Spectroscopic Characterization of Pantolactone in Water and DMSO Solutions

**Figure 1:**
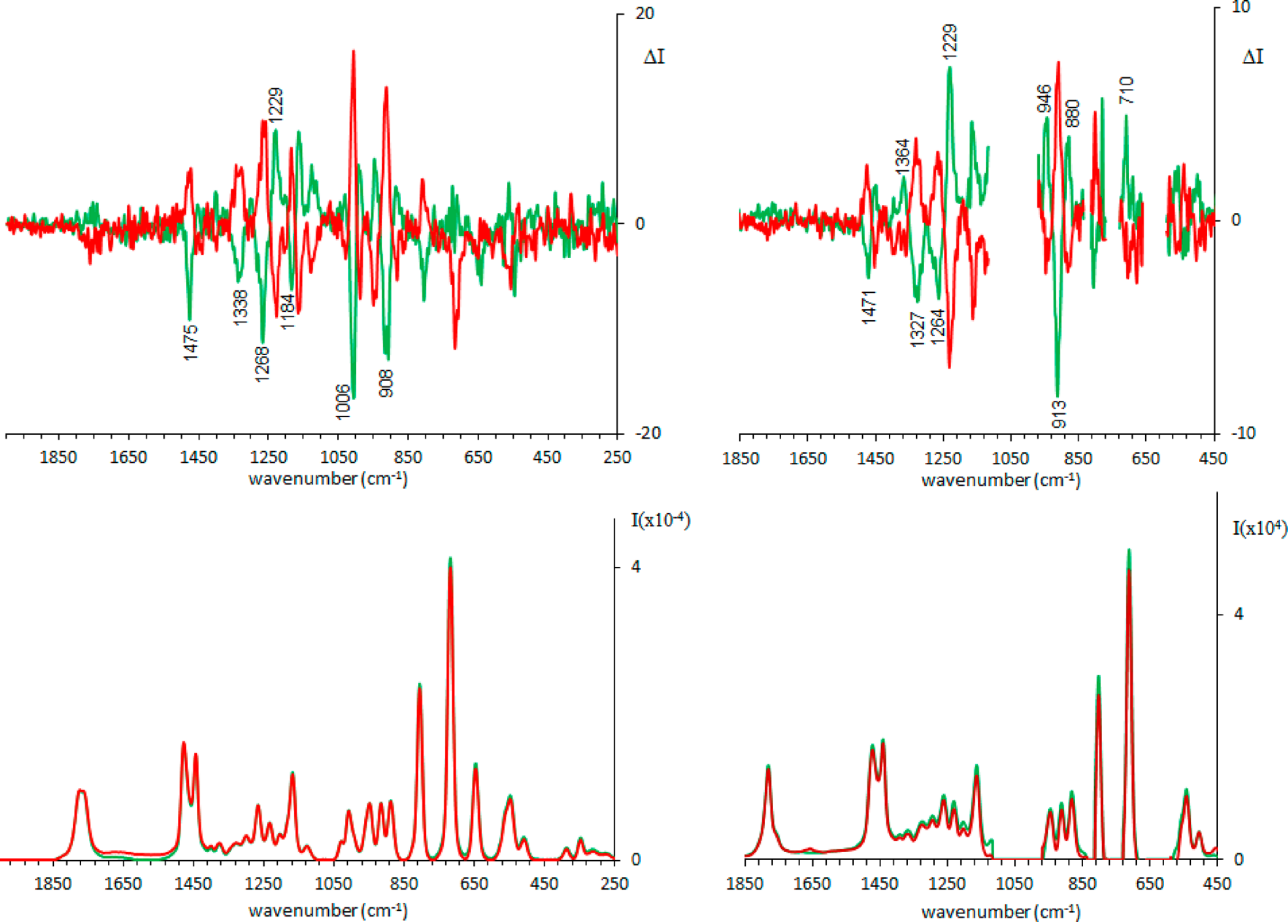
Experimental ROA (top) and Raman (bottom) spectra of (*R*)-pantolactone (green line) and (*S*)-pantolactone (red line) in water (left) and in DMSO (right).

**Figure 2:**
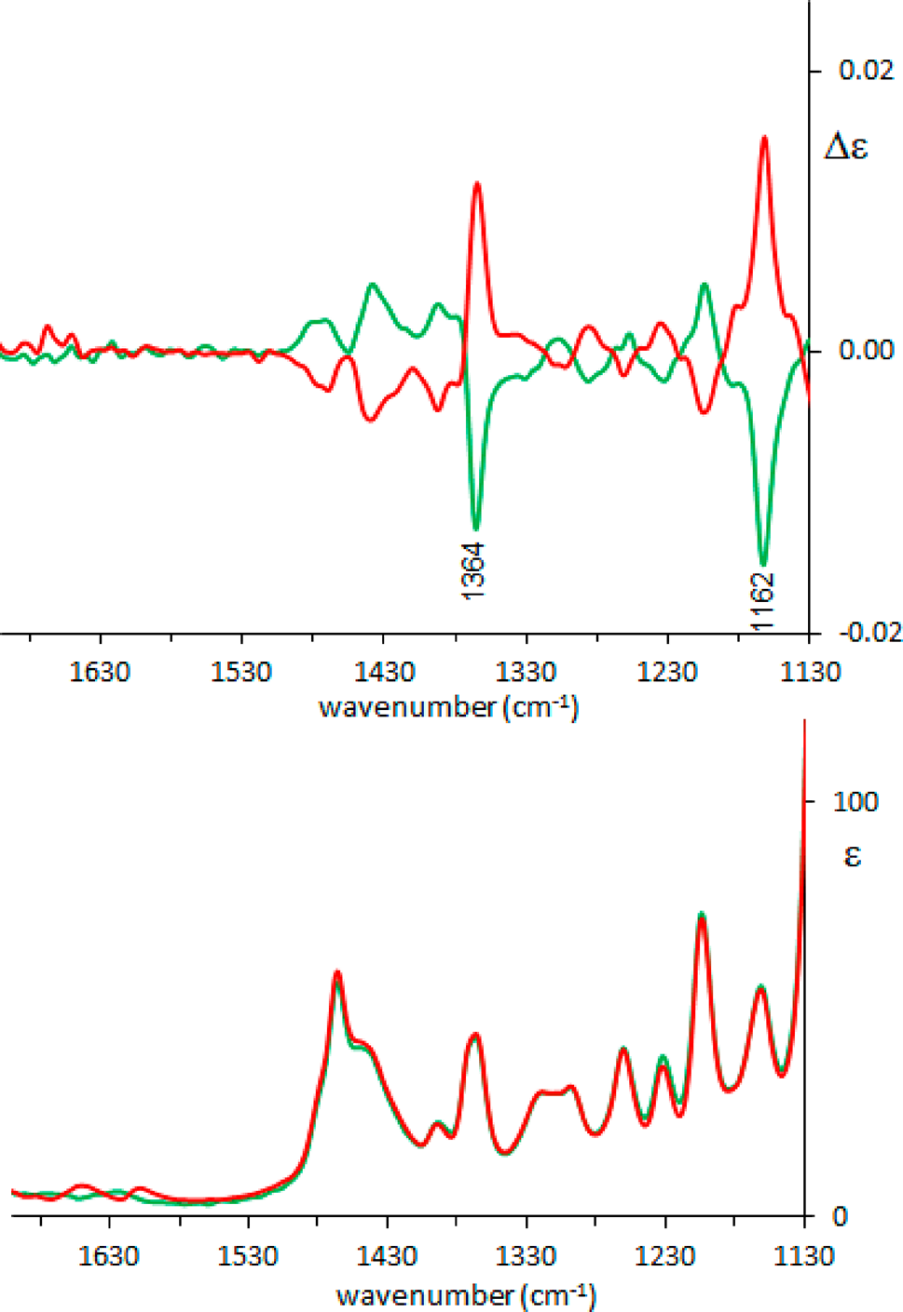
Experimental VCD (top) and IR (bottom) spectra of (*R*)-pantolactone (green line) and (*S*)-pantolactone (red line) in DMSO-*d*_6_.

We observe from Figure 1 that Raman and ROA spectra in the two solvents, water and DMSO, are quite similar: in the case of DMSO the ROA feature at 1229 cm^–1^ is more intense than the nearby bands, while the negative band at 1184 cm^–1^ observed in H_2_O is absent in DMSO (see Figure S1 for a better comparison). In both solvents one expects monomeric pantolactone molecules and strong solute–solvent interactions through H-bonds: water presents both acceptor and donor properties, while DMSO acts just as acceptor. In the second solvent also IR and VCD spectra have been collected (Figure 2).

### MD Simulations of Pantolactone in Water

In order to reproduce the observed data, a first analysis has been conducted by adopting the PCM approximation for the solvents and by calculating the spectra on the five most populated conformers obtained after conformational search in vacuo (see Table S1). One may observe that the dominant ring structure is _3_E-type envelope with the carbon atom bearing the two methyl groups, (see Scheme 1) outside the ring plane. We will show that spectra calculated at this level of theory give already an acceptable match with experiment (due to the low conformational mobility); however due to the strong solute–solvent interactions, one cannot be content with a study based just on PCM. For this reason, MD calculations have been conducted and analyzed in order to clarify solute–solvent interactions. Conformational changes observed during MD simulations take place on a much shorter time scale than the simulation time, so we are quite confident that we have explored the whole available conformational space with reliable statistical sampling: this is confirmed also by the fact that the CV values have reached a stable distribution (see Figure S2). Considering solute–solvent interactions, the radial distribution function *g*(*r*) reported in Figure S3 suggests strong interactions of water with the carbonyl oxygen and with the hydroxyl group; this has a correspondence with H-bond distribution, as evaluated from the frames of the MD trajectories, based on the criteria defined in the methods section. In particular, while the ring oxygen is H-bonded to water for 13% of the frames, the carbonyl forms one H-bond with water with 60% probability and two H-bonds with 11% probability. For 82% simulation time the hydroxyl group interacts with water as a donor and for 50% as acceptor.

### Calculation of ROA and Raman Spectra of Pantolactone in Water

**Figure 3:**
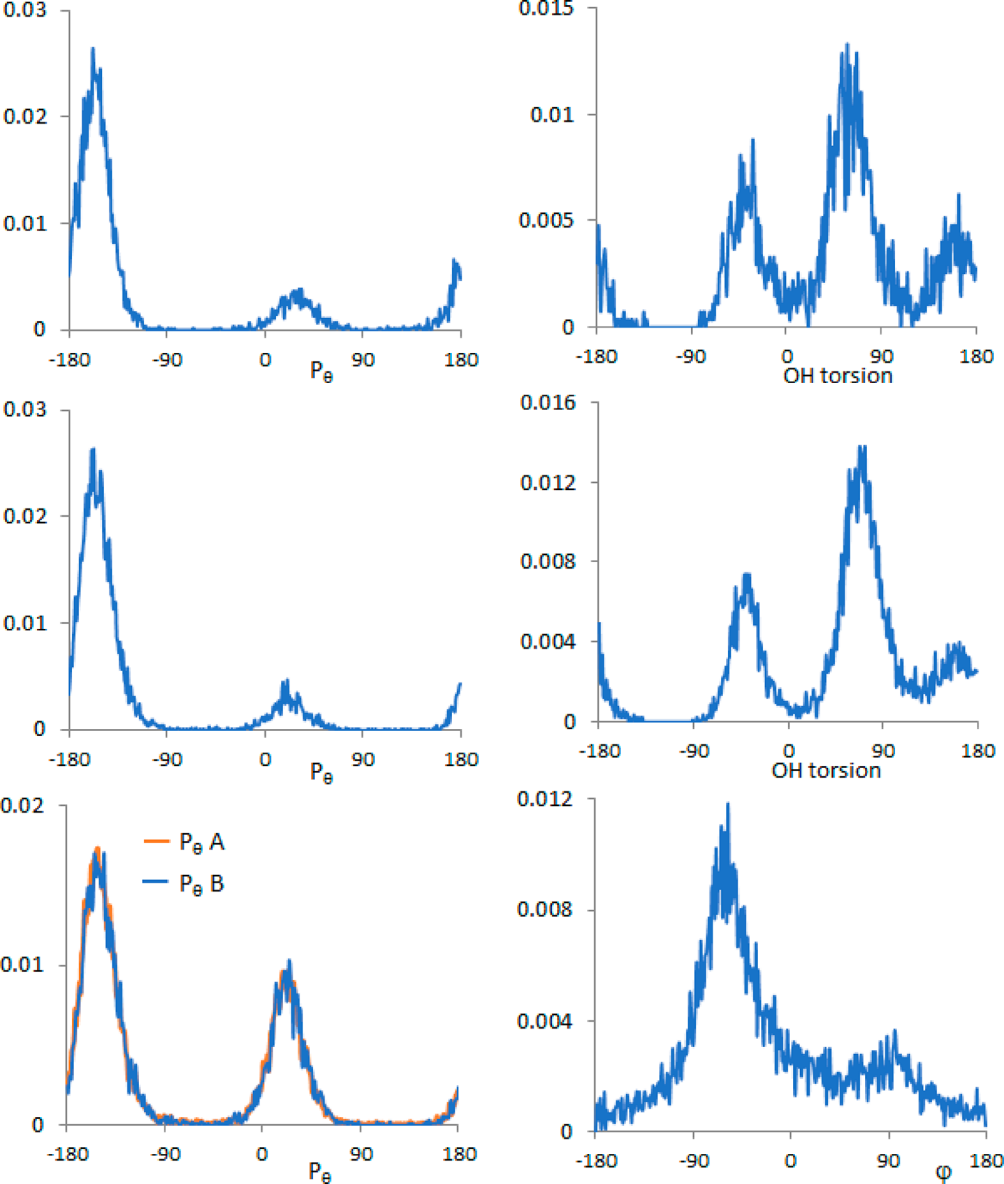
(*R*)-Pantolactone. Statistical distribution of the two collective variables, phase angle (*P*_θ_) of five-membered ring puckering (left) and OH torsion (right), for water simulation (top) and DMSO simulation (middle). Statistical distribution of the collective variables, phase angles *P*_θ_, eq 1, of the two five-membered ring puckerings (left) (*P*_θ_A for one ring (red) and *P*_θ_B for the other (blue)) and phase angle (φ) of the six-membered pseudo-ring (right), for CCl_4_ simulation (bottom). See refs (44) and (39) for the definition.

The previous analysis of MD simulations permits an adequate choice of the trajectory frames from the MD calculations to use in subsequent spectra calculations. On the basis of the statistical distributions of the two CV (dihedral hydroxyl angle and puckering phase) (Figure 3, top), cluster analysis has been conducted giving the results (population and geometrical characteristics) reported in Table 1. The explicit presence of the solvent somehow changes the OH dihedral angle average values and conformer populations with respect to what was obtained by PCM calculations. In any case, the ring conformation is similar in the two treatments.

**Table 1:** Population Factors and Values of Collective Variable *P*_θ_ and HOCC Torsion of Statistical Clusters of (*R*)-Pantolactone in Water

cluster	population (%)	*P*_θ_ (deg)	OH torsion (deg)
1	47.4	–157.3	81.2
2	22.1	–157.3	–37.2
3	10.0	18.7	–39.0
5	9.9	–157.6	149.2
4	8.0	20.9	–179.5
6	2.6	15.7	152.3

In order to calculate the spectra, the cluster representatives must be optimized; the solvent effect on pantolactone structure is preserved, either considering a solvent shell treated in the MM approximation (QM/MM) or considering a reduced shell treated at the same QM level for pantolactone and solvent molecules (QM/QM). Some tests have been conducted to examine the variability of the calculated spectra by considering many different snapshots at fixed time intervals within the same cluster in order to judge how reliable it is to take just the representative structure of the cluster. This indicates (see Figure S4) how some bands are particularly “robust” with respect to different yet similar structures while other features change upon little perturbations.^48,49^ The analysis shows that the most intense bands are well accounted for by the adopted clustering operation. In Figure S5, the comparison is also made between calculated spectra with shell water molecules treated explicitly in the normal-mode analysis and calculated spectra with shell water molecules frozen in the vibrational analysis.

**Figure 4:**
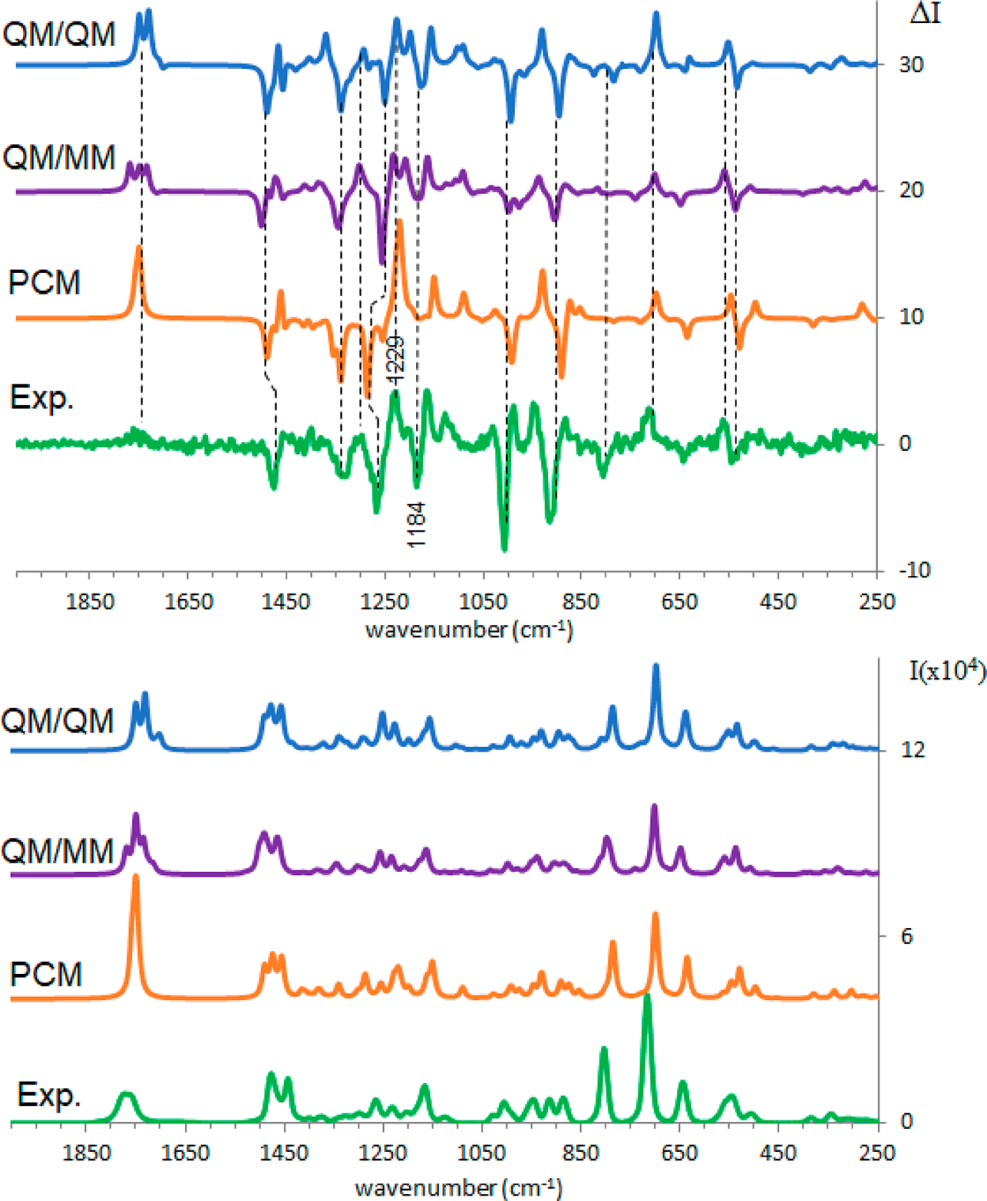
Comparison between experimental and calculated ROA (top) and Raman (bottom) spectra of (*R*)-pantolactone in water: green, experiment; orange, weighted average over conformers in PCM approximation; purple, weighted average over cluster representatives calculated with ONIOM method; blue, weighted average over cluster representatives calculated with explicit QM solvent. For all the calculations, 0.98 scaling factor was applied.

In Figure 4 the most significant calculated results are reported, showing that the best matching between theory and experiment is obtained with a reduced solvent shell treated at the same QM level as the molecule (QM/QM), while a more extended shell treated at the MM level does not provide advantages. The three calculated results reported in Figure 4 for appropriate comparison with experimental data are the following: (i) calculated spectra obtained after in vacuo conformational search, subsequent PCM optimization, and Boltzmann weighted conformational average (see Table S1 for details); (ii) weighted average spectra calculated on six cluster representatives obtained after MD simulation (weights given by the cluster populations) and ONIOM treatment, precisely with the solute at QM level and a 6 Å solvent shell (about 50 water molecules) treated at the MM level (QM/MM); (iii) weighted average spectra calculated on the same six cluster representatives in which a reduced 4 Å solvent shell has been considered (containing approximately 10–12 water molecules), with QM optimization of both solute and shell solvent molecules (QM/QM) however excluding solvent molecules from the subsequent vibrational analysis.

As anticipated above, the PCM calculations are already acceptable in comparison with the results obtained after MD analysis, while the QM/MM calculations are not fully satisfactory, especially as regard to ROA. The QM/QM calculation is definitely better: it predicts all the ROA experimental features with good relative intensities, representing the 1229 cm^–1^ band better in intensity than the PCM approximation and also correctly predicting the 1184 cm^–1^ negative band, which is associated with CH_2_ twisting coupled with stretchings of the ring simple CO-bonds, being the ring oxygen an HB-acceptor for nearby water molecules; furthermore the QM/MM and QM/QM calculations suggest a broad, low intensity feature for the carbonyl.

### MD Simulations of Pantolactone in DMSO

Also according to DMSO simulation, the two pantolactone molecules are predicted not to interact with each other but they show strong interaction with the solvent: H-bond analysis shows that 90% of the time the pantolactone OH is H-bonded to the solvent. Also in this case the same CV variables can be adopted to compare the structures and for clustering. The CV distributions (Figure 3, top and middle) are quite similar in the two solvents. As for the water case, cluster analysis permits extraction of six clusters, whose characteristics are reported in Table 2 (see also Table S2 for conformers obtained after in vacuo conformational search and PCM optimization).

**Table 2:** Population Factors and Values of Collective Variable *P*_θ_ and HOCC Torsion Clusters of (*R*)-Pantolactone in DMSO

cluster	population (%)	*P*_θ_ (deg)	OH torsion (deg)
**5**	49.9	–162.6	70.9
**1**	24.1	–158.4	–39.6
**3**	12.9	–161.3	153.2
**2**	5.7	16.7	49.1
**4**	4.9	13.9	83.1
**6**	2.5	16.0	–43.9

### Calculation of VOA Spectra of Pantolactone in DMSO

**Figure 5:**
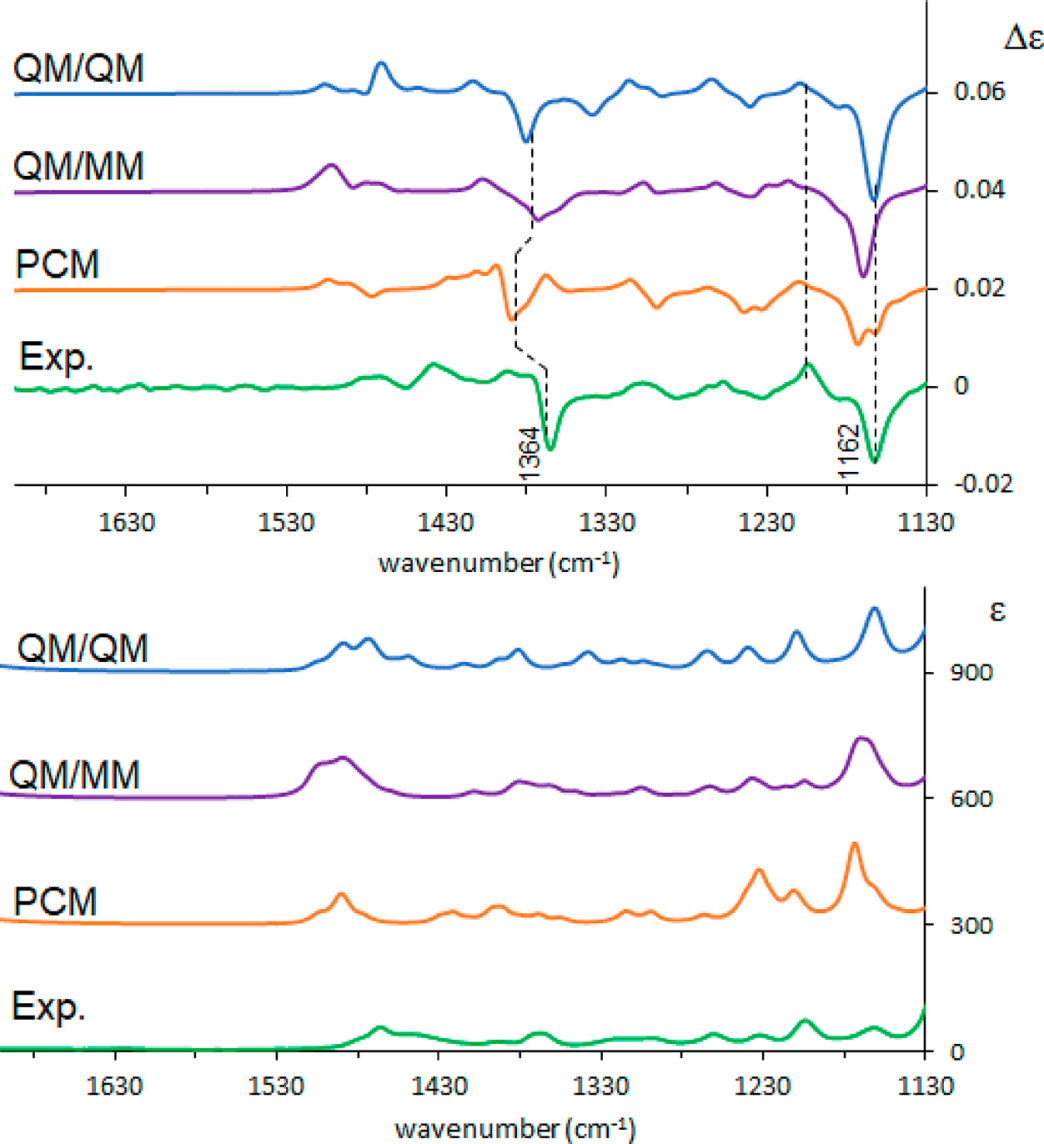
Comparison between experimental and calculated VCD (top) and IR (bottom) spectra of (*R*)-pantolactone in DMSO: green, experiment; orange, weighted average over conformers in PCM approximation; purple, weighted average over cluster representatives calculated with ONIOM method; blue, weighted average over cluster representatives calculated with explicit QM solvent. A 0.99 scaling factor was applied for PCM and QM/QM, 0.98 for QM/MM.

Subsequent to the analysis of the MD trajectory, both VCD and ROA spectra have been calculated, following the same three methods as before: standard PCM and Boltzmann weighted spectra after in vacuo conformational search; QM/MM calculated spectra of the cluster representatives; QM/QM for the same cluster representatives (in this case the shell has 4 Å radius and contains 6–8 DMSO molecules, mostly in the vicinity of the OH bond). The results are reported in Figure 5. The last choice gives the best match with the VCD experiment: it better reproduces the 1364 cm^–1^ band, which corresponds to C*H and OH bending vibrations, and also the 1162 cm^–1^ band is better predicted.

**Figure 6:**
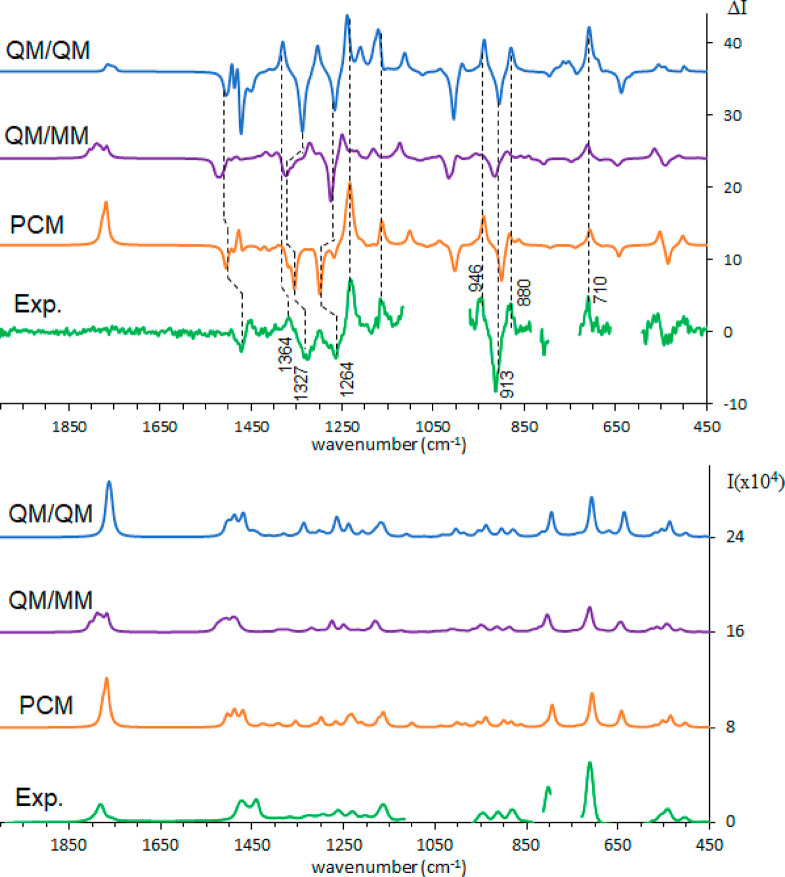
Comparison of experimental and calculated ROA (top) and Raman (bottom) spectra of (*R*)-pantolactone in DMSO: green, experiment; orange, weighted average over conformers in PCM approximation; purple, weighted average over cluster representatives calculated with ONIOM method; blue, weighted average over cluster representatives calculated with explicit QM solvent. A 0.99 scaling factor was applied for PCM and QM/QM, 0.98 for QM/MM.

Also, the ROA spectrum is well accounted for (see Figure 6). As pointed out above, the ROA spectra in water and DMSO are quite similar, and standard PCM calculations already give an acceptable result. The best improvement, considering the QM/QM case, concerns the carbonyl signal which gets weakened due to the average over the various geometries. The positive band centered at 710 cm^–1^ in the ROA spectra gains intensity in the QM/QM approach. The (+, −, +) triplet at 880, 913, and 946 cm^–1^ shows better relative intensity of the three bands, and also the three features with (−, −, +) signs at 1264, 1327, and 1364 cm^–1^ are better represented. From Figure S1, one may appreciate how the differences between the ROA spectra recorded in the two solvents, observed in the range 1150–1250 cm^–1^, are well reproduced by the QM/QM calculations, especially the band observed at 1184 cm^–1^ in water.

### Spectroscopic Characterization of Pantolactone in CCl_4_

**Figure 7:**
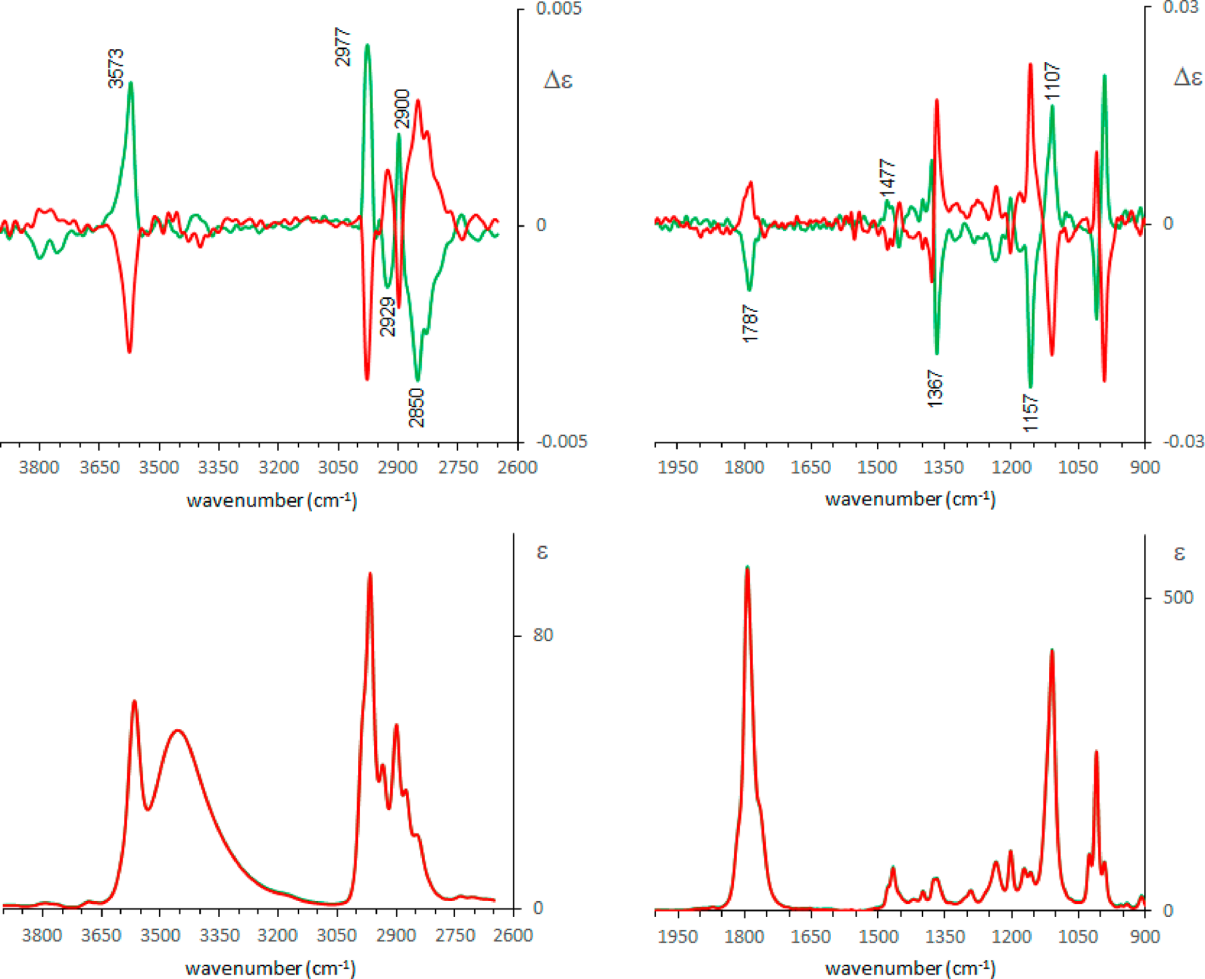
Experimental VCD (top) and IR (bottom) spectra of (*R*)-pantolactone (green line) and (*S*)-pantolactone (red line) in CCl_4_ solution.

VCD spectra of (*R*)- and (*S*)-pantolactone have been recorded also in carbon tetrachloride, not only in the mid-IR region but also in the CH and OH stretching regions (Figure 7). The mid-IR VCD spectrum presents two intense features at 1157 and at 1367 cm^–1^ (both negative for (*R*)-pantolactone) quite similar to the ones observed in the DMSO solution; only a few details of the weaker features in the 1230–1300 cm^–1^ range and at about 1450 cm^–1^ present some differences. It is important to recall that the first evidence of dimer formation had been provided by considering the CO and OH stretching absorption band;^20^ for this reason, we decided to evaluate IR and VCD spectra not only considering the PCM approximation subsequent to a standard MM conformational search but also taking into account MD simulation results. Due to the different solvent properties (no H-bond interactions with the solvent), the QM/MM approach is sufficient in this case.

### MD Simulations of Pantolactone in CCl_4_

MD simulations show the high propensity of pantolactone molecules toward dimerization. In the adopted simulative conditions, with the used force field, the dimer forms within a few ns (also starting from different initial conditions). The two molecules then separate from each other, and then they dimerize again such that the dimeric form is present for 85% of the simulation time. The dimer, although stabilized by intermolecular hydrogen bonds, is quite flexible.

Three CVs were chosen to analyze the trajectory structures: two five-membered ring puckering *P*_θ_ variables (one for each pantolactone ring) and the phase φ of the six-membered ring puckering^39,44^ relative to the “pseudo” ring bridging the two pantolactone molecules. This last CV allows one to describe the dimer assembly held together by the H bonds. Considering Scheme 1, the six atoms defining the intermolecular ring are 6–1–8–6′–1′–8′. The time evolution of this CV during MD simulations is an indication of the flexibility of the dimeric structure (Figure S6). In Figure 3 (lower part) the distribution for the three CV is reported: the distribution function of the six-membered ring puckering has an absolute maximum in correspondence with the value −64° providing a boat-type pseudo-ring, while the secondary maximum gives a skew-type pseudo-ring (90°). As far as the usual five-membered ring puckering is concerned, the distributions calculated on the two molecules coincide, as expected: the two envelope conformations with the carbon bearing the two methyl groups either above or below the ring plane are the most populated, similar to what was found in the monomeric case in water and DMSO solvents; here the two ring conformers are similarly populated. By use of the k-medoids algorithm^38,43^ on the three distributions, 16 clusters have been obtained (see Table 3). Regarding intermolecular H-bonds, two of them are present between the two molecules for 56% of the simulation, just one for 29% of simulation time.

**Table 3:** Population Factors and Values of Collective Variable^a^ and OH Torsions for Ring A and Ring B

cluster	population (%)	*P*_θ_A (deg)	*P*_θ_B (deg)	φ (deg)	OH torsion A (deg)	OH torsion B (deg)
**10**	12.7	–157.3	–156.0	–76.1	77.0	74.3
**6**	10.2	–149.0	14.1	–45.8	74.5	72.5
**14**	8.9	–159.6	20.5	–57.7	65.2	68.1
**2**	8.7	21.6	–154.2	–58.1	65.1	61.7
**12**	8.0	–149.1	–149.2	93.7	76.8	79.5
**8**	7.9	12.4	–143.7	–58.4	89.4	90.6
**4**	6.9	–146.4	–144.2	–129.6	79.9	80.0
**15**	6.7	–152.1	–152.5	–27.6	61.7	64.5
**3**	5.4	–146.3	–143.7	143.8	89.6	85.7
**9**	4.1	–152.0	–155.0	30.5	39.5	40.7
**1**	3.9	–149.3	20.1	43.9	83.3	79.5
**7**	3.8	21.7	25.9	–36.1	71.6	74.1
**16**	3.3	30.6	–148.0	51.5	68.8	63.0
**5**	3.3	11.1	–143.2	62.4	77.2	57.5
**13**	3.1	–13.5	7.9	–36.4	53.4	84.4
**11**	3.0	7.1	17.8	–40.2	74.0	77.9

a Two phase angle of puckering of the two five-membered ring *P*_θ_ of ring A and ring B and phase of pseudo-six-membered ring puckering φ.

### Calculation of VCD and IR Spectra of Pantolactone in CCl_4_

**Figure 8:**
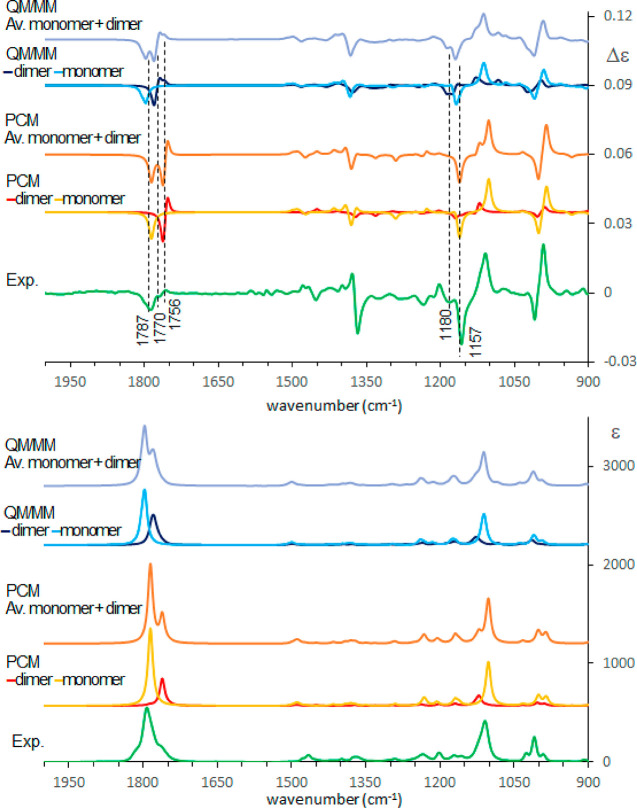
Comparison of experimental and calculated VCD (top) and IR (bottom) spectra of (*R*)-pantolactone in CCl_4_: green, experiment; red, yellow, and orange, standard PCM calculations; dark blue, blue, and light blue, MD–ONIOM calculations. 0.985 wavenumber scaling factor has been applied. The monomer and the dimer contributions are obtained after a weighted average of the different conformers based on Table 3, Table S3, and Table S4 and have been scaled based on dimer/monomer populations determined at 50 mM concentration considering ref (20).

As for the other cases, preliminary calculations with the solvent considered at the PCM level have been carried out obtaining similar structures as the one previously reported^21^ for the monomer and for the dimer (see Tables S3 and S4 for conformer characterization). Literature data^20−22^ show that important contributions from dimers should be considered, being responsible inter alia for the observed optical rotation value. In Figure 8 we present the calculated results at this level of approximation: the mid-IR spectrum is well accounted for by the monomer. We also note that the negative VCD band for the carbonyl stretching had been already observed and explained in other monomeric (*R*)-γ-butyrolactones.^50^ However, the weak VCD features at about 1180 and 1756 cm^–1^ (indicated in Figure 8) and the shape of the IR carbonyl and OH stretching bands suggest that dimers may be important. The calculated spectrum of the dimer at the PCM level, while still confirming many of the monomer bands, shows the presence of a sharp doublet in the carbonyl region, which, though, is not observed.

Considering then results from the cluster representatives obtained with MD simulations, also for the monomeric regime (in this case, using the same CV adopted in the two previously examined solvents), we have calculated the spectra after optimization of the solute with a CCl_4_ shell treated at the MM level (QM/MM). In the mid-IR region, as shown in Figure 8, the IR and VCD spectra obtained with this protocol are very similar to those obtained with the simple PCM calculation. We observe however that the weighted average relative to dimers with the QM/MM treatment exhibits a smoothed carbonyl doublet when averaged over the statistically representative structures. Then, after further averaging of the monomer and the dimer spectra, based on the equilibrium constant taken from ref (20), there is a considerable improvement in the prediction of IR and VCD band-shape. The two protocols, namely, conformational search + PCM and MD simulation + ONIOM, give similar results for the principal features but differ in the shape of bands which is better predicted by QM/MM after MD analysis.

**Figure 9:**
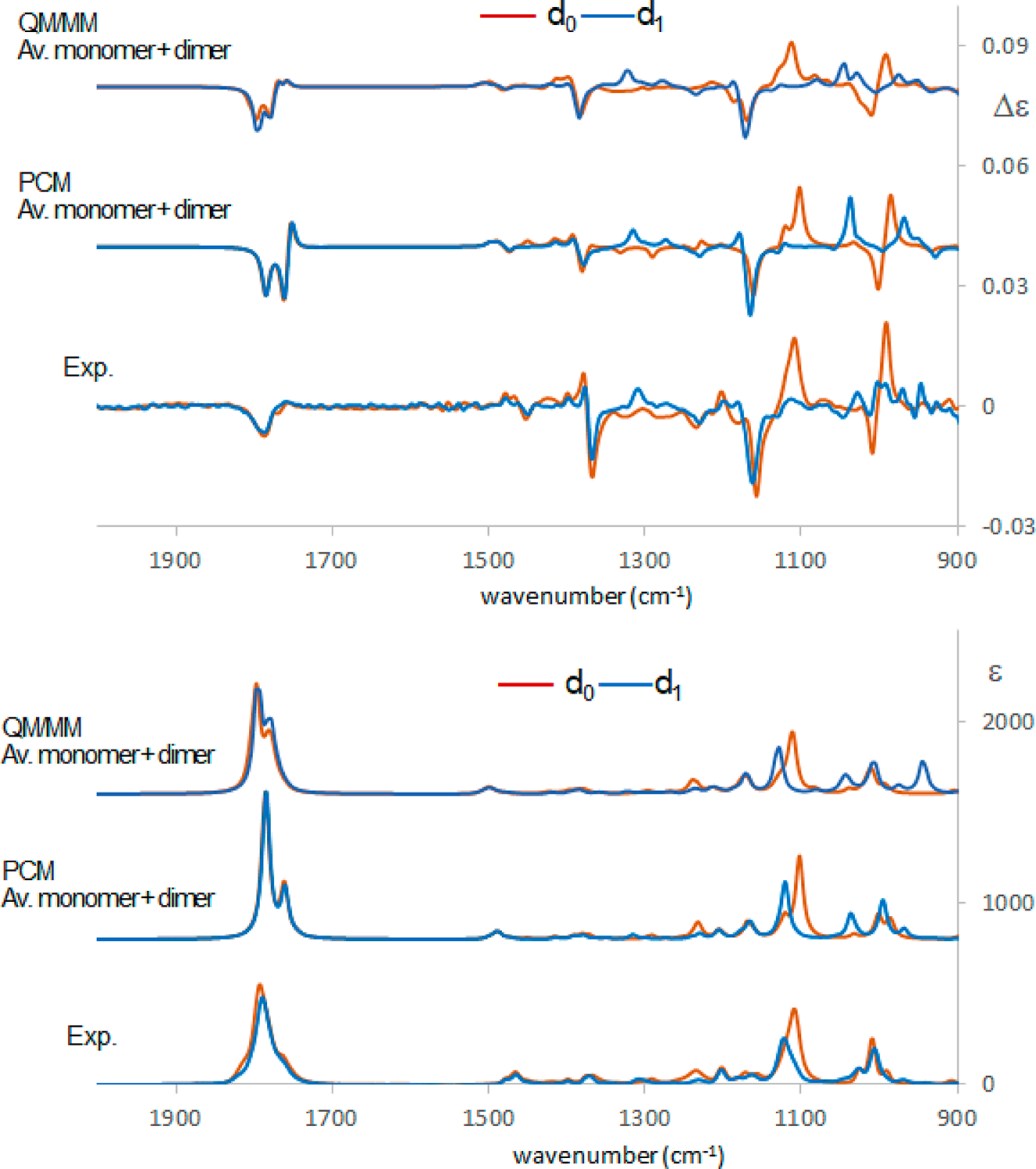
Comparison of experimental and calculated VCD (top) and IR (bottom) spectra of (*R*)-pantolactone-d_0_ (brown) and (*R*)-pantolactone-d_1_ (blue) in CCl_4_: bottom traces, experiment; middle traces, standard PCM calculations; top traces, MD–ONIOM calculations. 0.985 wavenumber scaling factor has been applied. The monomer and the dimer contributions are obtained after weighted average of the different conformers based on Table 3, Table S3, and Table S4 and have been summed after scaling based on dimer/monomer populations determined at 50 mM concentration considering ref (20).

In order to confirm whether the adopted QM/MM protocol is appropriate also for a (slightly) different case, we considered pantolactone with a deuterated hydroxyl group and recorded the mid-IR VCD spectrum in CCl_4._ In Figure S7, analogous to what done in Figure 8, we compare experimental IR and VCD spectra with calculated ones both in the PCM and in the QM/MM approaches for both dimer and monomer. The carbonyl stretching features exhibit the same behavior as observed for the hydrogenated species; the mid-IR region shows some differences due to changes of normal modes involving either the OH or the OD group: in the deuterated case, it is more difficult to pinpoint bands that are diagnostic of the presence of the dimer in solution. In Figure 9 the comparison hydrogenated/deuterated species is shown both for the experiment and for the calculations. Once again the QM/MM calculation appears to better reproduce the data. In particular, the peak calculated at 1035 cm^–1^, which is predicted to be quite sharp in the PCM approximation, is broadened according to QM/MM representation, in agreement with experimental data.

The same type of calculation has been used for the high wavenumber range, comprising the OH and CH stretching regions. The spectra in the OH stretching region should in principle discriminate between monomer and dimer. This is true for absorption. For VCD instead only a monosignate band is observed at 3573 cm^–1^, which can be assigned to free OH stretching, while no bands are observed (within the sensitivity limits of our experiment) in correspondence of the broad absorption band at 3450 cm^–1^, despite the fact that dimeric structures generate huge calculated VCD doublets (see Figure S8). Due to the sharp, intense calculated OH stretching absorption and VCD doublet, the stable dimers predicted by PCM cannot account for the observed broad IR features.

**Figure 10:**
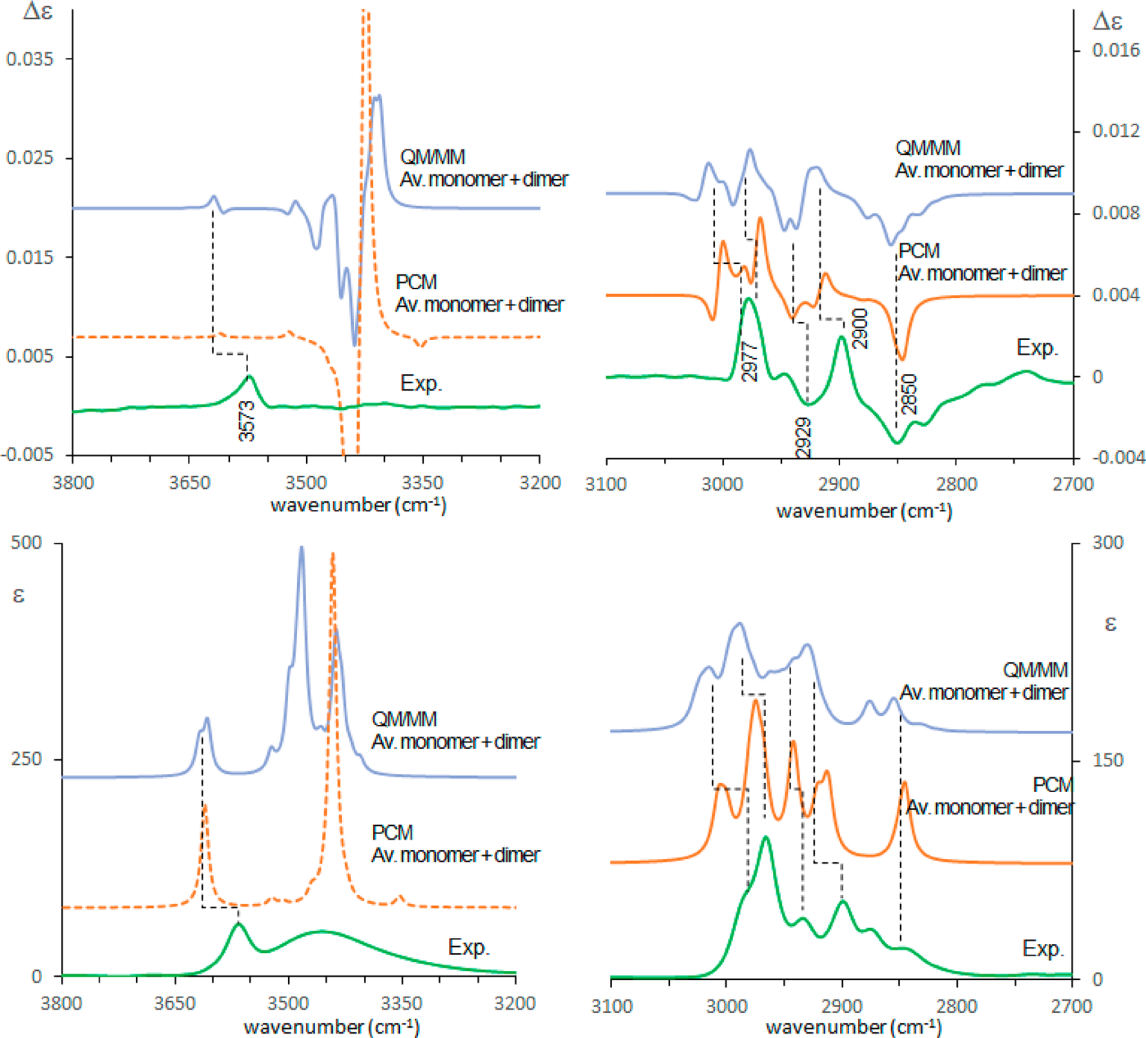
Comparison of experimental and calculated VCD spectra of (*R*)-pantolactone in CCl_4_ in the OH and CH stretching region: green, experiment; orange, standard PCM calculations; light blue, MD–QM/MM calculations. The monomer and the dimer contributions are obtained after weighted average of the different conformers based on Table 3, Table S3, and Table S4 and have been summed after scaling based on dimer/monomer populations determined at 50 mM concentration considering ref (20). 0.96 wavenumber scaling factor has been applied.

As expected, the calculated features of the CH stretching region are not very sensitive to dimerization but seem quite sensitive to the presence of the explicit solvent shell since in this case the band shape improvement is evident (see Figure 10, with details of the spectra obtained for the dimers and monomers given in Figure S8). In the OH stretching region, the band due to the monomeric form is better represented in intensity and shape by the QM/MM procedure (Figure 10). Considering dimer contributions, the simple PCM calculations give sharp and huge features, while the QM/MM procedure suggests the broadening effect. However, the use of just the representative structures of the clusters seems not sufficient to account for the smoothed and broad observed IR band and of the fact that no appreciable VCD activity is recorded. The difficulty is due also to the fact that the features of each single structure are really very strong (see Figure 10 and Figures S8 and S9).

In conclusion, even though the contribution of monomers is proven by the ∼3573 cm^–1^ IR and VCD feature, the presence of dimers appears evident from the broad IR absorption at ∼3400 cm^–1^, as already well-known; however, if one wishes to construct a satisfactory model for the hydroxyl stretching regions, the number of optimized conformers is deemed to be fairly large. As a general result, the calculations presented here clearly show that in the case of CCl_4_ the inclusion of the solvent shell, treated simply by MM, can really improve the band-shape and suggest how calculated OH stretching intense signals are “nonrobust” with respect to solvent perturbation justifying the experimentally weak or absent VCD signal.

**Figure 11:**
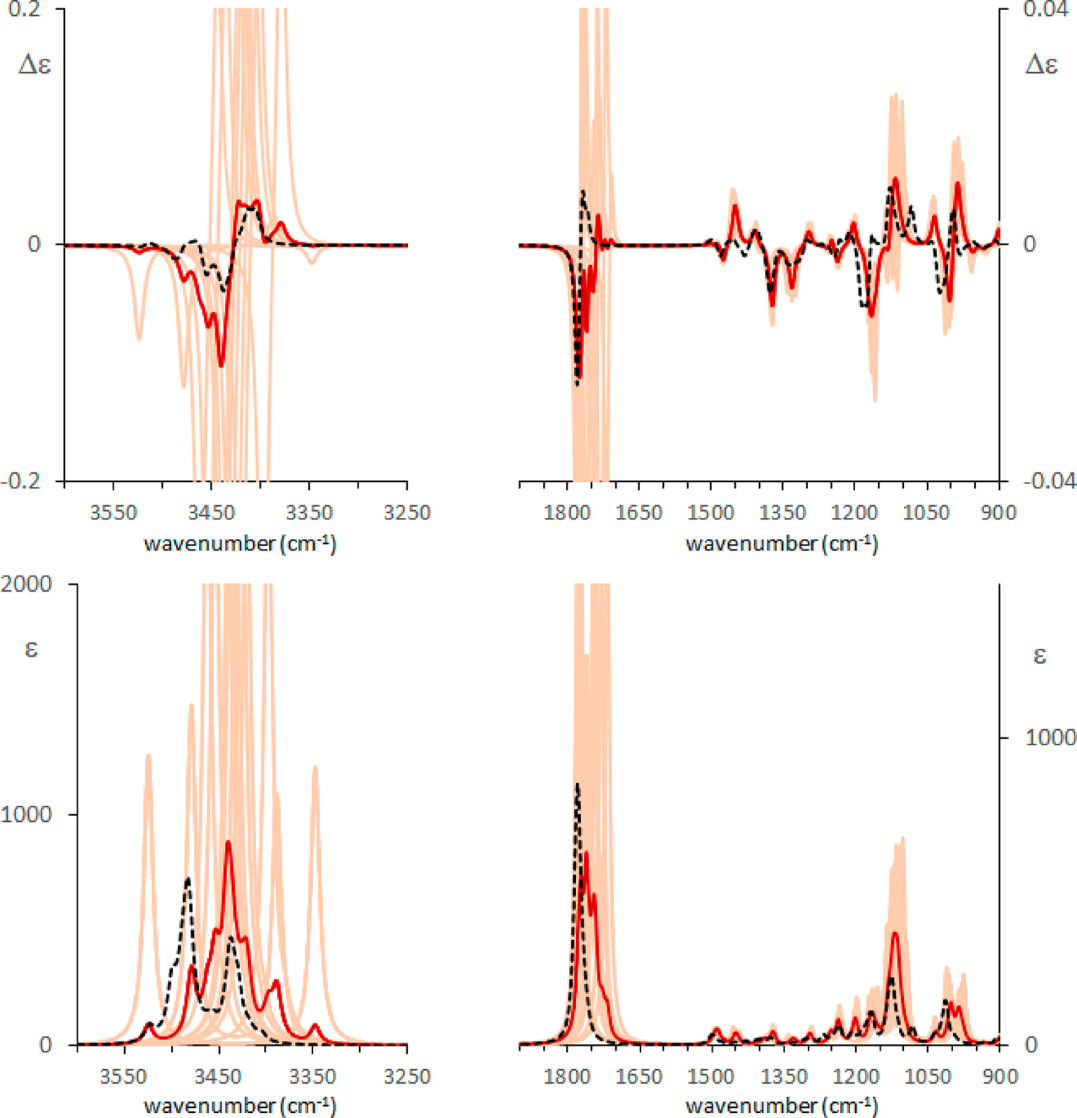
PCM calculated IR (bottom) and VCD (top) spectra of (*R*)-pantolactone in CCl_4_ on 12 structures obtained from the most populated conformer displaced along the three lowest wavenumber normal modes. The red trace is obtained upon Boltzmann weighted average over such spectra. The black broken trace is the one obtained upon averaging over the 16 clusters optimized with a MM solvent shell as previously commented. 0.96 wavenumber scaling factor has been applied to the OH stretching region, 0.985 to the mid-IR region.

A further test has been conducted for this instance, keeping in mind that the effect of the explicit solvent is to perturb the structure optimized in a solvent continuum model. A way to monitor the influence onto the spectra due to perturbations on the minimum geometry is to distort in little steps the dimeric system along with the lowest energy normal modes, i.e., “intermolecular” vibrations. A similar method indicating VCD sensitivity to large-amplitude/low-frequency modes was adopted in the case of intramolecular degrees of freedom for floppy molecules.^51−53^ It has already been observed that some bands can be particularly sensitive to little structural changes, even promoting sign reversal and showing great intensity and frequency variations. We have considered here the most populated dimeric conformer, obtained after simple PCM optimization, and have displaced the structure along with the lowest energy normal modes, which are distortions of the intermolecular H-bonded bridge. By reporting in Figure 11 the superposition of spectra calculated in this way, one may appreciate how displacements along large amplitude normal modes may weaken and broaden very intense features. The average spectra obtained in this way compare well with the average over the representatives of the MD clusters.

## Conclusions

In this work, pantolactone has been characterized by VCD and ROA spectra in protic and nonprotic solvents; the set of new data has led us to use this molecule as benchmark to test various protocols for computation of spectroscopic responses in different environments and allowing also for aggregation. In particular, after careful conformational search, standard PCM calculations have been conducted and the results compared with MD analysis followed by QM/MM or QM/QM calculations (within IEF-PCM frame), the last term meaning that pantolactone and solvent molecules within the solvent shell defined by MD were treated at the same QM level. Such methods had been already partially tested in the literature, particularly in the case of water solutions, but treatment of organic solvents is less common. An important point is the criterion for how to choose snapshots from simulations to calculate the chiroptical response. The choice is to go for either a large number of snapshots at fixed time intervals (in the hypothesis that the simulation is long enough and there are no high barriers among different conformational basins) or for representative structures at conformational minima identified by previous analysis of the most significant dihedral angles^3^ or, finally, for representative structures chosen with a cluster analysis usually referred to rmsd calculation. In the present cases we adopted statistical cluster analysis based on significant collective variables adapted to the considered system, an idea borrowed from metadynamics protocols.^54,55^ Here, we have relied on ring puckering coordinates also in the case of intermolecular ring comprising H-bonds. After the snapshot selection, the DFT optimization method has to be chosen: how much extended the solvent shell, which approximation to adopt for the solute and the shell, and so on. In this work, for solvents with H-bond donor or acceptor atoms exhibiting high H-bond propensity like water and DMSO we obtained good results considering a limited shell treated at the same QM level as the solute (herein called QM/QM). In the case of CCl_4_ the shell can be treated at the MM level adopting a ONIOM procedure (herein called QM/MM); the presence of the solvent and the variety of structures taken from MD simulations help to obtain good band-shapes. In the case of dimers the procedure permits taking into account perturbations of the solvent on flexible dimeric structures and thus avoiding (or at least limiting) of artifacts due to the fixed structures proposed by PCM of the solute dimer alone. Comparison with spectra calculated on structures displaced along low-frequency/large-amplitude normal modes is helpful.

The proposed spectra calculations encompass IR and VCD data in regions more extended than the usual mid-IR, which is generally not extremely sensitive to H-bond interactions, unless H-bonds heavily influence the conformational landscape of the solute, which is not the case for pantolactone. The carbonyl region is more informative in the IR band-shape and in the presence or absence of VCD signals. The less common OH and CH stretching regions are also examined: good results are obtained for band-shape of the OH absorption bands and for CH stretching VCD and absorption features, despite the fact that anharmonicity has been disregarded in our calculations.^56−58^ Difficulties are encountered in the VCD OH stretching region, where huge features, highly dependent on minimal structure variations, are calculated: further studies are needed to improve the statistics suggesting how this usually disregarded spectroscopic region is quite challenging for theoretical calculations. Also in this respect we feel that anharmonicity treatment should be undertaken in future work in order to check the influence on IR and VCD intensity.

## Supporting Information Available

Tables providing geometrical parameters and population factors for (*R*)-pantolactone monomer and dimer calculated at the IEFM-PCM DFT level; Figures S1, S4, S5 containing calculated Raman and ROA spectra (in the mid region) in water and DMSO elaborating information from the text; Figure S7 reporting comparison of calculated mid-IR VCD and IR spectra of deuterated d_1_ species in CCl_4_; Figure S8 and S9 reporting comparison of calculated VCD and IR spectra in the OH stretching region; Figures S2, S3, and S6 containing the time dependence and statistical analysis of relevant variables for (*R*)-pantolactone monomer and dimer (PDF)
