# Supplementary material for: Solvent Effects and Aggregation Phenomena Studied by Vibrational Optical Activity and Molecular Dynamics: The Case of Pantolactone
Source: J Phys Chem B. 2020 May 12;124(22):4512–26. doi: 10.1021/acs.jpcb.0c01483 (PMC8007093; doi:10.1021/acs.jpcb.0c01483)
Supplement: Supplementary file 1 — jp0c01483_si_001.pdf [file jp0c01483_si_001.pdf]

Solvent Effects and Aggregation Phenomena Studied by Vibrational Optical Activity and Molecular Dynamics: the Case of Pantolactone

Simone Ghidinelli[1], Sergio Abbate[1,2], Jun Koshoubu[3], Yasuyuki Araki[4], Takehiko Wada[4], Giovanna Longhi[1,2]

[1]*Dipartimento di Medicina Molecolare e Traslazionale, Università di Brescia, Viale Europa 11, 25123 Brescia (Italy)*
[2]*Istituto Nazionale di Ottica (INO), CNR, Research Unit of Brescia, c/o CSMT via Branze 45, 25123 Brescia (Italy)*
[3]*JASCO Corporation, 2967-5 Ishikawa-machi. HachiojI, Tokyo 192-8537, Japan*
[4]*Institute of Multidisciplinary Research for Advanced Materials, Tohoku University, 2-1-1 Katahira, Aoba-ku, Sendai, Miyagi 980-8577, Japan*

LIST OF FIGURES AND TABLES

1) Figure S1. Superimposed Experimental and QM/QM Calculated ROA spectra for (*R*)-pantolactone in water and DMSO-d_6.
2) Table S1. (*R*)-pantolactone monomer: Population and CV values of the conformers calculated at IEF-PCM in water.
3) Figure S2. Left: Statistical distribution of phase angle of 5-membered ring puckering of (*R*)-pantolactone in water. Right: Time evolution of phase angle of 5-membered ring puckering of (*R*)-pantolactone during MD simulations.
4) Figure S3. Radial distribution functions of the atoms of (R)-pantolactone participating in the hydrogen bond with water.
5) Table S2. (*R*)-pantolactone monomer: Population factors and CV values of the populated conformers calculated at IEF-PCM in DMSO.
6) Table S3. Population factors and CV values of the populated conformers of pantolactone monomer in CCl_4 calculated at IEF-PCM level.
7) Table S4. Population factors and CV values of the populated conformers of pantolactone dimer in CCl_4 calculated at IEF-PCM level.
8) Figure S4. Calculated ROA and Raman spectra of (*R*)-pantolactone in water for ten snapshots belonging to the most populated cluster.
9) Figure S5. Comparison of the QM/QM calculated ROA and Raman spectra of the weighted average of clusters of (*R*)-pantolactone in water with the experimental one.
10) Figure S6. Time evolution of the Collective Variable φ during MD simulations for (*R*)-pantolactone dimer in CCl_4.
11) Figure S7. Experimental and calculated VCD and IR spectra of (R)-pantolactone-OD in CCl_4.
12) Figure S8. Comparison of experimental and calculated VCD (top) and IR (Bottom) spectra of (R)-pantolactone in CCl_4 in the OH and CH stretching region.
13) Figure S9. Calculated VCD spectra of the representative of the 16 clusters in CCl_4.

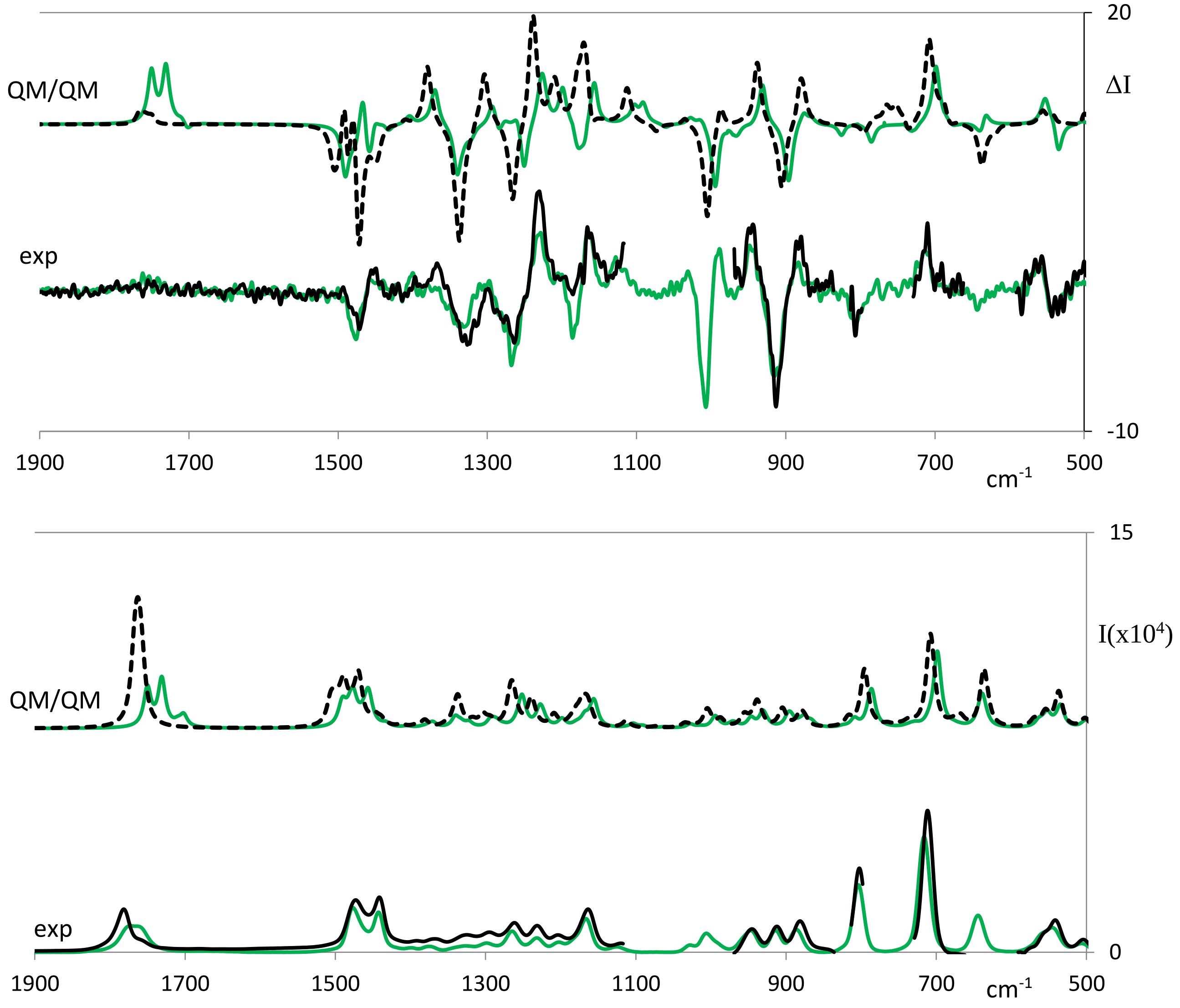

Figure S1. Comparison between experimental and calculated ROA (top) and Raman (Bottom) spectra of (*R*)-pantolactone in water (green trace) and DMSO (black trace). Calculated spectra are weighted average over cluster representatives calculated with explicit QM solvent. Scaling factors: 0.98 for water case, 0.99 for DMSO case.

Table S1. Population and CV values of the conformers calculated at IEF-PCM in water. 3D structure of the five populated conformers are shown below the table.

Conformer	*pop%*	*OH torsion (deg)*	*P_θ (deg)*	*Conformation*
1	68.3	40	-173.	$_3E$
2	25.7	154	-169	$_3E$
3	4.2	68	19	^3E
4	1.5	168	18	^3E
5	0.4	-60	13	^3E

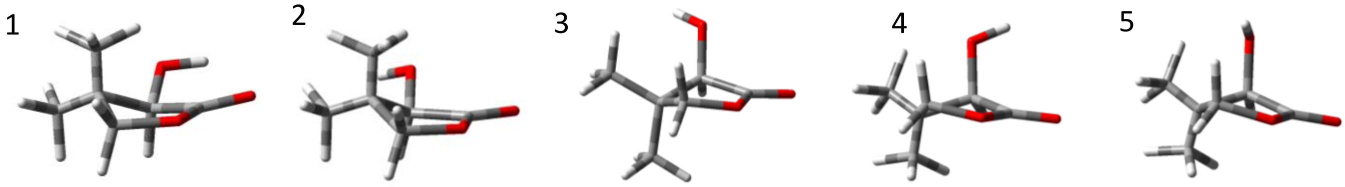

In case of vacuum calculation on the first conformer is populated.

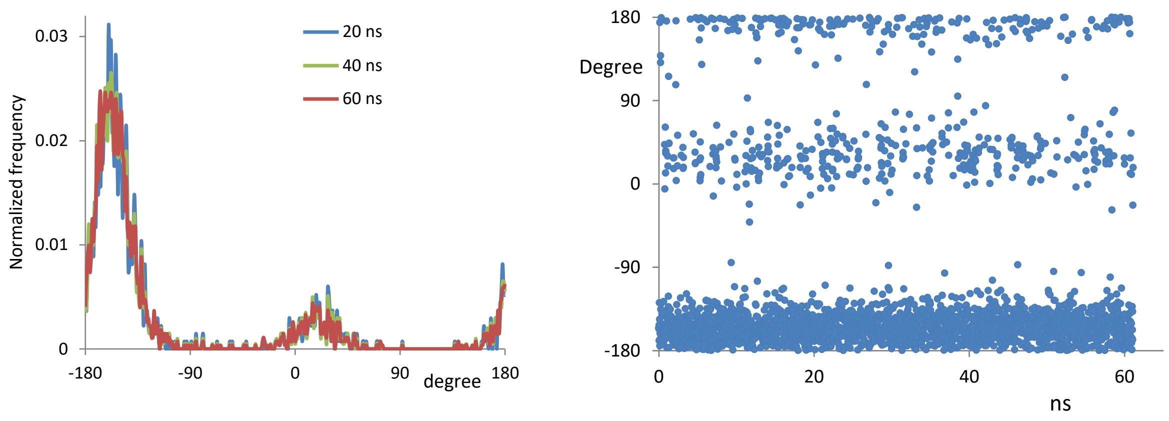

Figure S2. Left: Statistical distribution of phase angle of 5-membered ring puckering of (*R*)-pantolactone in water. Right: Time evolution of phase angle of 5-membered ring puckering of (*R*)-pantolactone during MD simulations

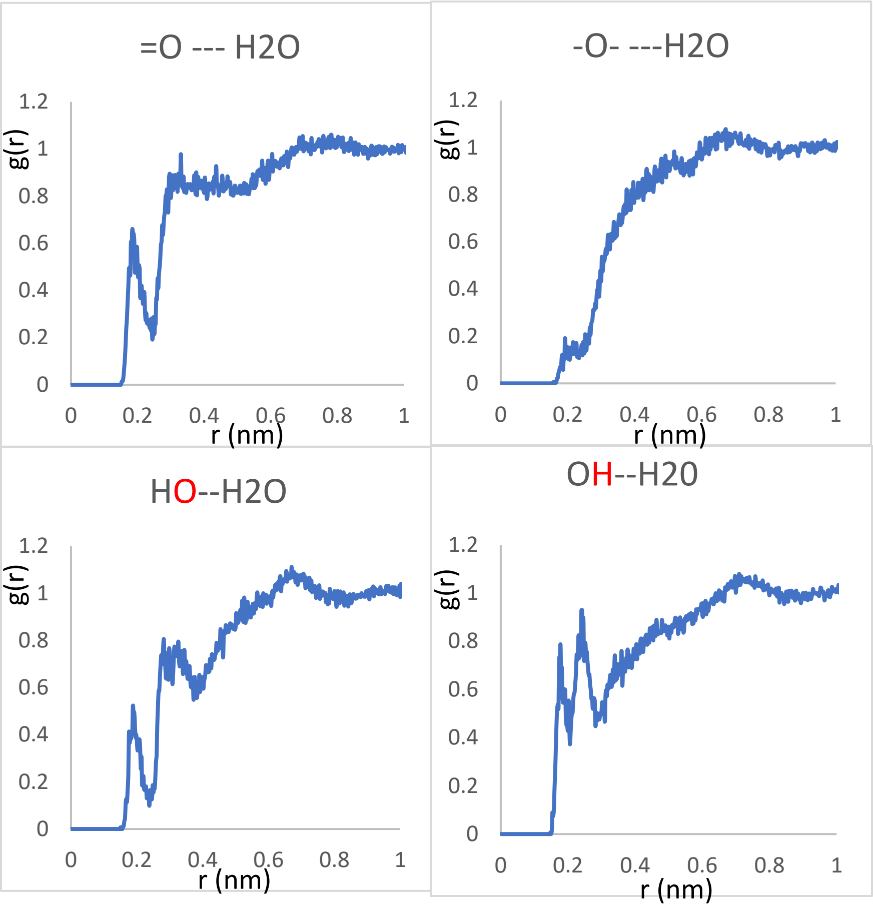

Figure S3. Radial distribution functions of the atoms of (*R*)-pantolactone participating in the hydrogen bond with water.

Table S2. Population factors and CV values of the populated conformers calculated at IEF-PCM in DMSO

conformer	pop %	OH torsion(deg)	P_θ (deg)	*Conformation*
1	70.4	32	-169.	$_3E$
2	23.9	155	-166.	$_3E$
3	4.3	68	15.	^3E
4	1.4	168	18.	^3E

Table S3. Population factors and CV values of the populated conformers of pantolactone monomer in CCl_4 calculated at IEF-PCM level

conformer	pop %	OH torsion(deg)	P_θ (deg)	*Conformation*
1	95.8	25	-170	$_3E$
2	4.2	61	18	^3E
3	0.0	-49	17	^3E

Table S4. Population factors and CV values of the populated conformers of pantolactone dimer in CCl_4 calculated at IEF-PCM level

conformer	pop%	**OH-A**	**OH-B**	**P_θA (deg)**	**P_θB (deg)**
1	*81.7*	83	83	-171	-171
2	*5.6*	91	67	-171	-170
3	*3.2*	-39	-39	-171	-171
4	*3.1*	-47	69	-171	10
5	*2.6*	83	88	-171	13
6	*1.8*	-47	73	-171	-169
7	*1.8*	-46	73	-171	-169

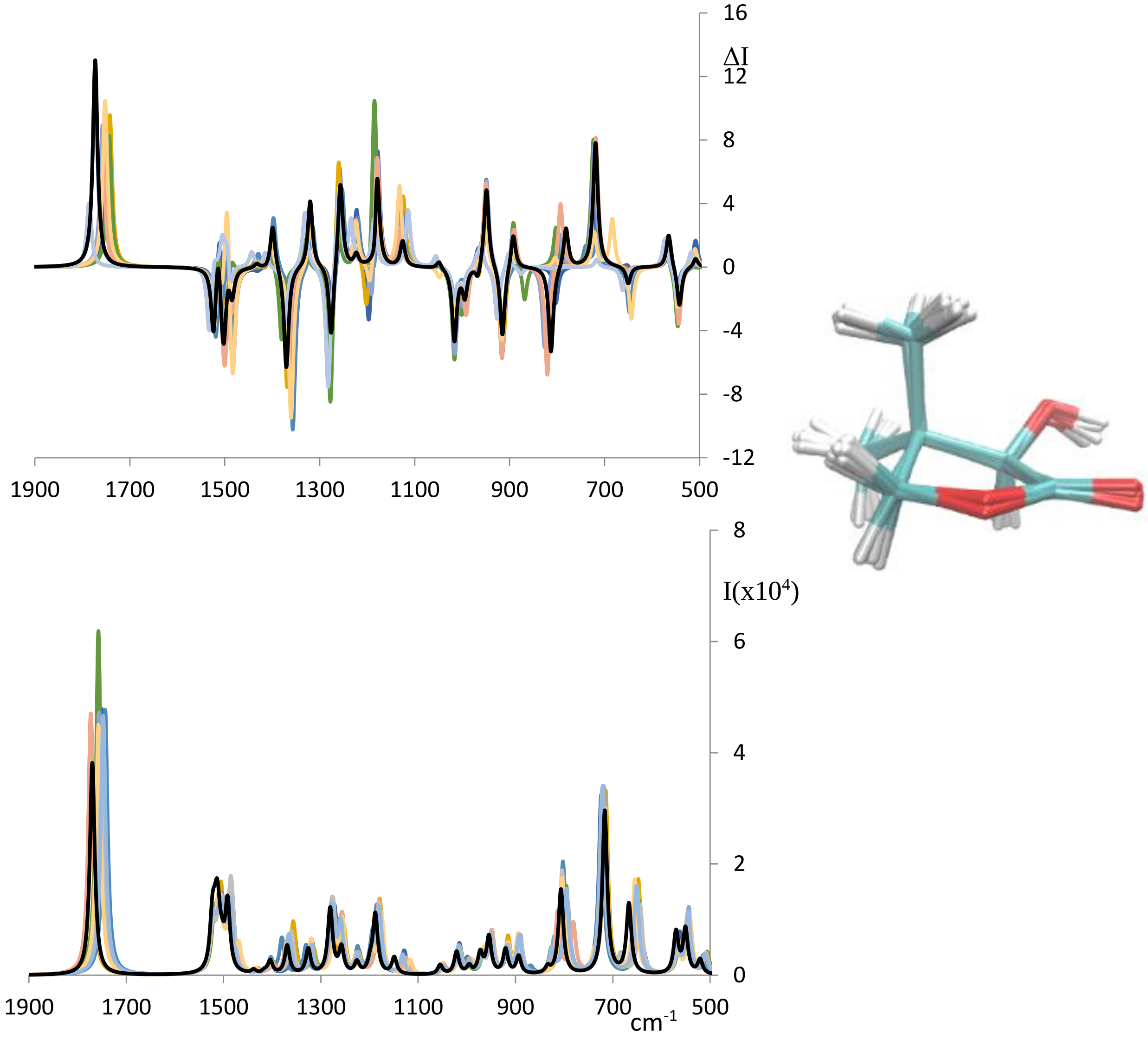

Figure S4. Calculated ROA and Raman spectra of (*R*)-pantolactone in water for ten snapshots belonging to the most populated cluster (colored lines) equally spaced in simulation time. The representative of the cluster is shown in black line. The corresponding superimposed structures are shown on the right side.

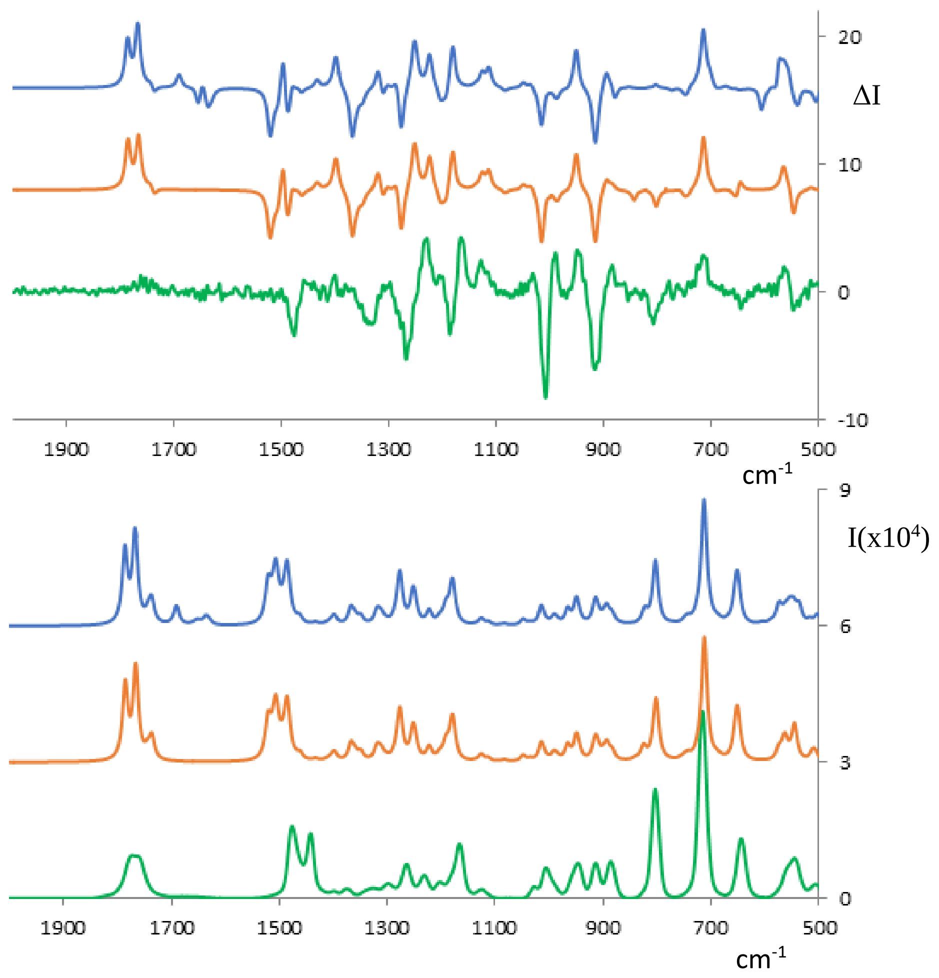

Figure S5. Comparison of the QM/QM calculated ROA (top) and Raman (bottom) spectra of the weighted average of clusters of (*R*)-pantolactone in water with the experimental one. Green: experimental; blue: QM/QM with shell water molecules optimized but frozen in the vibrational analysis; orange: QM/QM with all water molecules of the shell kept active.

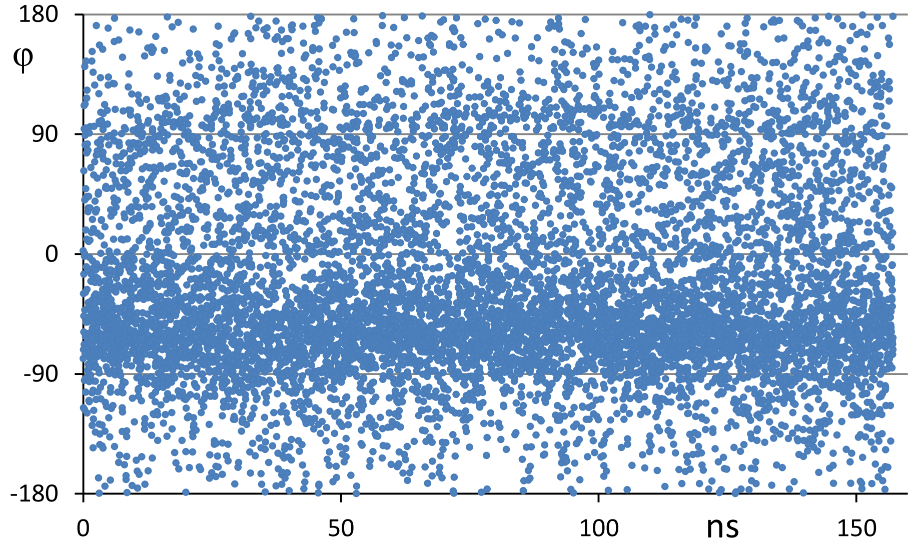

Figure S6. Time evolution of the Collective Variable φ (°) during MD simulations for (*R*)-pantolactone dimer in CCl_4.

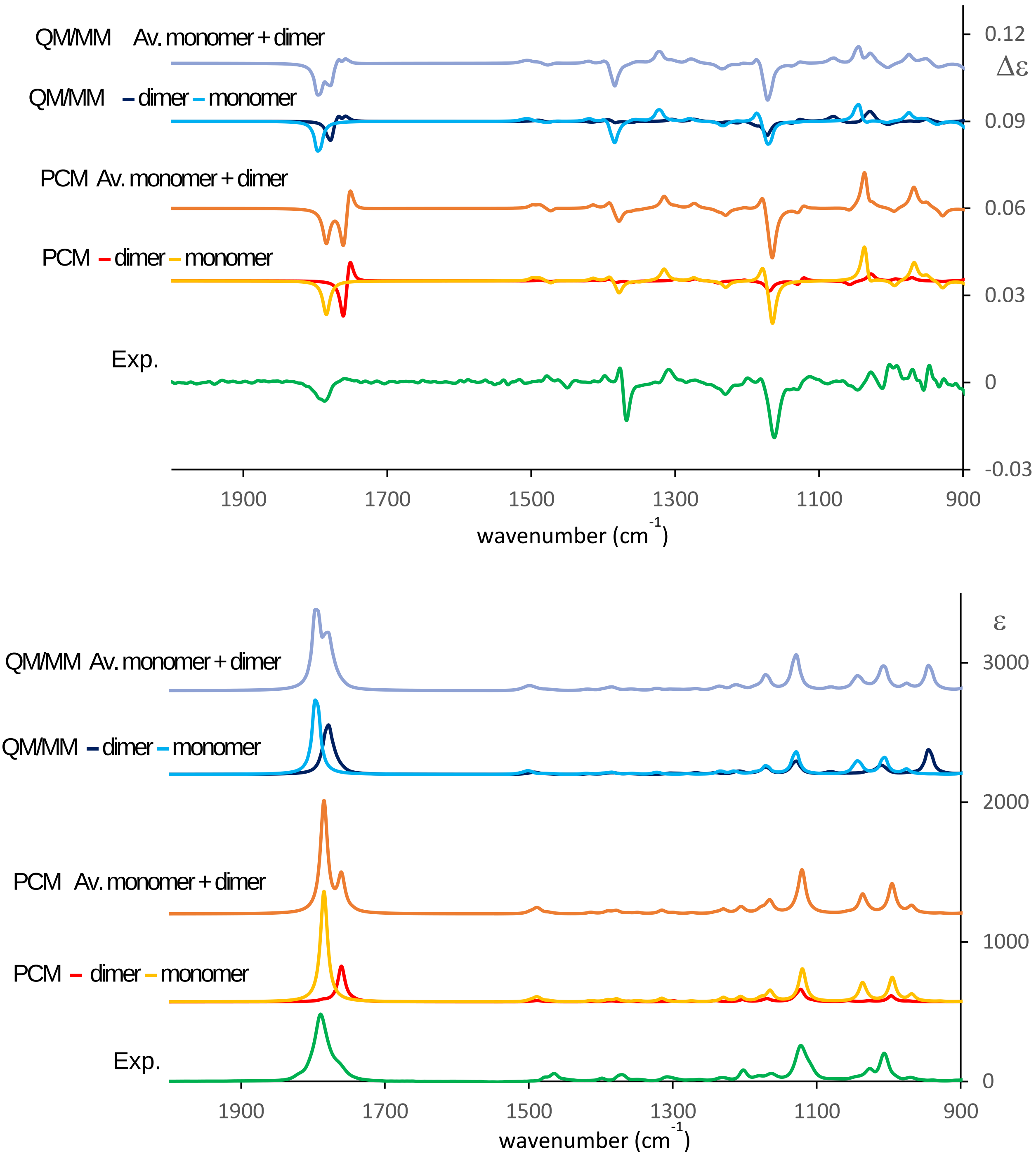

Figure S7. Comparison of experimental and calculated VCD (top) and IR (Bottom) spectra of (*R*)-pantolactone-d_1 in CCl_4. Green: experiment; red, yellow and orange: standard PCM calculations. Dark blue, blue and light blue: MD-ONIOM calculations. 0.985 wavenumber scaling factor has been applied. The monomer and the dimer contributions are obtained after weighted average of the different conformers based on Table 3 and Tables S3 and S4 and have been scaled based on dimer/monomer populations determined at 50 mM concentration considering reference: *Bull. Chem.Soc. Jpn.*, 1985, 58, 1767.

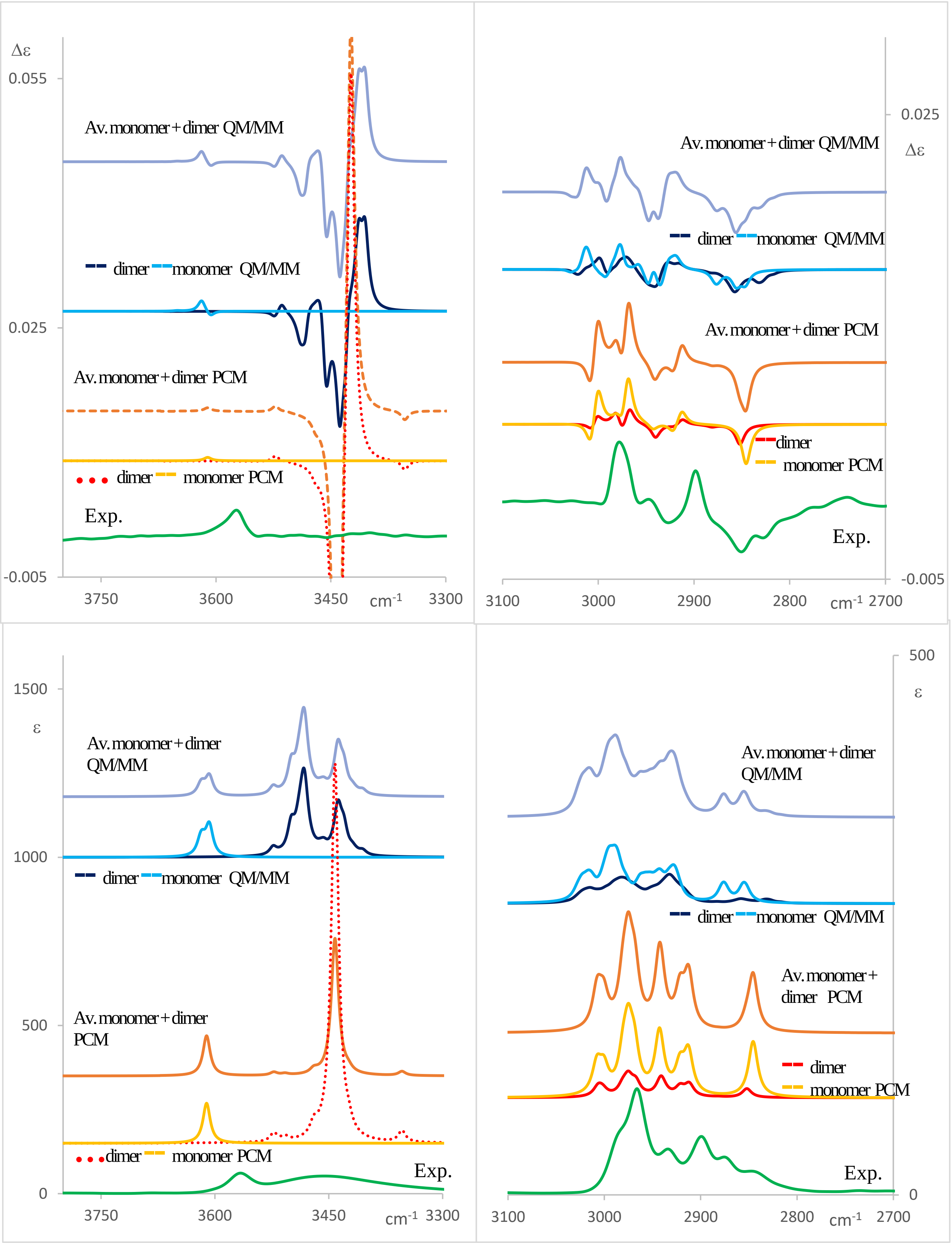

Figure S8. Comparison of experimental and calculated VCD (top) and IR (Bottom) spectra of (*R*)-pantolactone in CCl_4 in the OH and CH stretching region. Green: experiment. Red, yellow and orange: standard PCM calculations. Dark blue, blue and light blue MD-ONIOM calculations. 0.96 wavenumber scaling factor has been applied. The monomer and the dimer contributions are obtained after weighted average of the different conformers based on Table 3 and Tables S3 and S4 and have been scaled based on dimer/monomer populations determined at 50 mM concentration considering reference: *Bull. Chem.Soc. Jpn.*, 1985, 58, 1767.

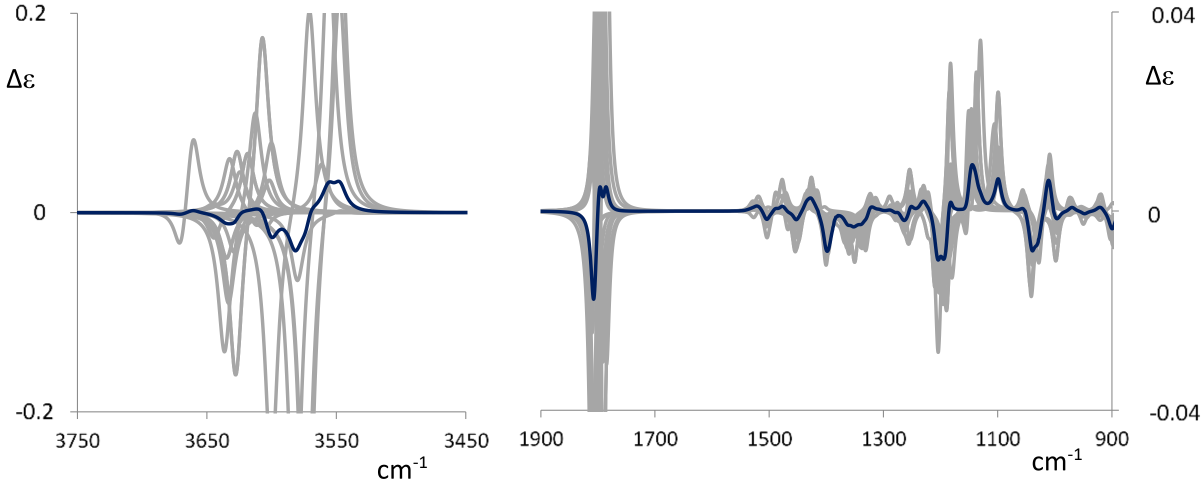

Figure S9. (*R*)-pantolactone in CCl_4: Calculated VCD spectra of the representative of the 16 clusters (grey lines) and their weighted average (blue line) in CCl_4
